# Abundance and Equality

**DOI:** 10.3389/frma.2022.977684

**Published:** 2022-12-01

**Authors:** Mauritz Kop

**Affiliations:** ^1^AIRecht, Amsterdam, Netherlands; ^2^School of Law, Stanford University, Stanford, CA, United States

**Keywords:** abundance society, post-scarcity economic theory, equality, good governance, technology, post-Rawlsian Equal Relative Abundance (ERA) principle of distributive justice, intellectual property, capitalism

## Abstract

The technology driven post-scarcity society is upon us. Ubiquitous technologies are eradicating scarcity in many industries. These macroscopic system trends are causing our economy to transition from relative scarcity to relative abundance. For many people in the world however, in both developed, developing, and underdeveloped countries, the notion of an Age of Abundance will sound utterly bizarre. There is a tension between abundance and equality. Good governance considers in what manner the state conducts public policy, manages public resources and promotes overall prosperity. This chapter connects good governance to the end of scarcity and integrates equality into abundance. The chapter critically examines the normative justifications of our scarcity based legal institutions, such as property and intellectual property (IP) systems, in light of 10 exponential, Fourth Industrial Revolution (4IR) technologies, and the post-scarcity economy. Starting point is that absolute and relative abundance are not utopian. Technology will erase scarcity in more and more economic areas in the foreseeable future, but not everywhere or for everybody. The chapter views relative scarcity and relative abundance as temporal socio-economic categories at two opposite sides of a continuum. The chapter unifies good governance with equality and abundance, by introducing a post-Rawlsian Equal Relative Abundance (ERA) principle of distributive justice. This includes defining a set of material and immaterial primary goods, warranting adequate, sufficient levels of relative abundance (which depend on technological evolution), and equitable results per region or group. Crucially, ERA integrates desert-based principles to the degree that some may deserve a higher level of material goods because of inequality in contributions, i.e., their hard work, talent, luck or entrepreneurial spirit, only to the extent that their unequal rewards do also function to improve the position of the least advantaged. A society governed by the ERA principle should in theory be able to solve the poverty trap on a global level. As lifting people from poverty in Europe is a different thing than achieving ERA in the US, applying equal relative abundance techniques in Asia and Africa each have their own specific challenges and dimensions.

*Truly I tell you*,

*whatever you did for one of the least of these brothers and sisters of mine*,

*you did for me*.

*Matthew 25:40, 45, NIV*

## One sentence synopsis

This chapter connects good governance to the end of scarcity and unifies equality with technology driven abundance, by introducing the Equal Relative Abundance (ERA) principle of distributive justice.

## Executive summary

1. The technology driven post-scarcity society is upon us. Ubiquitous technologies are eradicating scarcity in many industries. These macroscopic system trends are causing our economy to transition from relative scarcity to relative abundance. A shift to abundance concerns system wide changes on a regional, national and global level, that—in addition to the economy—also affect our socio-political institutions and our environment.
  For many people in the world however—in both developed, developing, and underdeveloped countries—the notion of an Age of Abundance will sound utterly bizarre and totally misplaced. There is a tension between abundance and equality.
2. Good governance considers in what manner the state conducts public policy, manages public resources and promotes overall prosperity. This chapter connects good governance to the end of scarcity and integrates equality into abundance. It provides suggestions on how resources and the means of production can be effectively managed in an affluent, “Cornucopian” society, with the aim of equitable outcomes for the masses instead of desirable results for select groups. The chapter critically examines the normative justifications of our scarcity based legal institutions, such as property and intellectual property (IP) systems, in light of 10 exponential, Fourth Industrial Revolution (4IR) technologies, and the post-scarcity economy.
3. Starting point is that absolute and relative abundance are not utopian. Technology will erase scarcity in more and more economic areas in the foreseeable future, but not everywhere or for everybody. This phenomenon is known as the poverty paradox. Considering that the social costs of inequality—such as a clear perception of social injustice, social exclusion, a decrease in productivity and health, and an increase in violence—are an important barrier to achieving widespread relative abundance conditions, the post-scarcity paradox must be resolved with priority.
  This chapter views relative scarcity and relative abundance as temporal socio-economic categories at two opposite sides of a continuum.
4. In addition, technological progress is often at odds with the law, in particular property law, antitrust law and IP. So how should the law and our legal institutions look like in a post-scarcity society? The way in which we design our systems of property, fair competition and IP influences many aspects of how our society operates. The same applies to the architecture of our technology. As IP and ownership arrangements shape technology, technology shapes IP. As society shapes its legal institutions, legal institutions (and traditions) shape society.
5. To put present day social transformation in its proper historical context, the chapter explains—from a bird's eye view—orthodox economic theory based on scarcity, the different phases of capitalism, the stages of development of government systems and the importance of the separation of powers (EU) as prescribed by Montesquieu's *trias politica*, or a system of checks and balances (US).
6. To shape and clarify our thinking about the transition from scarcity to abundance, we investigate whether ideas and theories of great philosophers and economists including Marx, Kant, Hegel, Hume, Mill, Keynes, Demsetz, Schumpeter, and Rawls are applicable to the structure and organization of society during the Age of Abundance. All this requires an open-minded approach.
7. Principles of distributive justice offer moral guidance for the political frameworks and legal institutions that influence the distribution of benefits, risks, rights and responsibilities across members of society. These frameworks and systems directly impact people's lives. In finding answers to the challenges that lay ahead of us, the chapter considers distributive justice principles and methods associated with utilitarianism, egalitarianism, welfare-theory, consequentialism, equality of opportunity, luck, responsibility and desert.
8. The chapter unifies good governance with equality and abundance, by introducing a post-Rawlsian Equal Relative Abundance (ERA) principle of distributive justice. This includes defining a proper set of material and immaterial primary goods, warranting adequate, sufficient levels of relative abundance (which depend on technological evolution), and equitable results per region or group. ERA builds on the difference principle and combines it with desert-based critique, while incorporating post-scarcity values and ideals that would make sense in our new context of relative sustainable abundance conditions. Crucially, ERA integrates desert-based principles to the degree that some may deserve a higher level of material goods because of inequality in contributions, i.e., their hard work, talent, luck or entrepreneurial spirit, only to the extent that their unequal rewards do also function to improve the position of the least advantaged.
9. The chapter views the concept of society through a broad, interdisciplinary lens. While framing key aspects and goals of present-day societies and describing their shift to a state of pervasive relative abundance, we can draw historical timelines of progressing forms of society. Society as a concept can be studied and defined from various scientific disciplines, such as political science, sociology, cultural anthropology, and philosophy. The abundance society concept consolidates these notions, as much as scientifically sound.
10. During the transition to the Age of Abundance, more and more forms of global governance will be put into operation, conceptually separating the abundance society from territoriality and from the nation state. And so, the abundance society evolves into a cosmopolitan, technologically advanced global human civilization. As a large, networked sphere in which Earth's regions and nations, and people's socio-cultural identities are united. In that sense, the abundance society is a macro model of a world system.
11. A society governed by the ERA principle should in theory be able to solve the poverty trap on a global level. During the transition to the abundance society, ERA will have to be operationalized in a differentiated way. As lifting people from poverty in Europe is a different thing than achieving ERA in the US, applying equal relative abundance techniques in Asia and Africa each have their own specific challenges and dimensions. In addition to an overarching vision, this irrevocably requires customization and experimentation. Datadriven, multimethod discussions should inform the final design and regionally optimized implementations of ERA.
12. The chapter argues the need for reform and reimagining existing legal institutes based on the philosophy of canonical thinkers, as well as doctrines such as the tragedy of anticommons, and concepts such as the post-work society and a new social contract based on equal relative abundance.
13. It then offers an overview of 10 disruptive 4IR key technologies that are rapidly propelling and shaping the transformation to a post-scarcity model. These are artificial intelligence, big data, quantum technology, nanotechnology, biotechnology, 3D printing, nuclear fusion, DLT/blockchain, virtual and augmented reality, and hyper-accurate positioning.
14. After that, the chapter links these technologies to policies that will enable conversion from the legacy economy to widespread relative abundance. It gives examples of the strategic reforms needed right now, in the midst of the 4IR, as well as reforms necessary during the Age of Abundance, tailored to specific industries, economic sectors and technologies. The chapter connects the method of technology forecasting to forecasting abundance and offers lawmakers concrete policy recommendations and pathways to the next phase.
15. An Age of Abundance requires a government system tuned for abundance. When thinking about such a system, we need to reconcile social, economic, and political theory, in light of the function and purpose of the state. The chapter looks at contemporary principles of distributed justice for answers, including the notion of the market as a self-correcting mechanism in concert with the equalizing effect of central planning, and government adjustments, such as taxes and antitrust regulation.
16. The chapter posits that it is urgent to start experimenting with prototypes of systems that mix the best parts of acceptable, forward thinking socialist and ethical post-capitalist paradigms, built on participatory democracy. When searching for a post-scarcity synthesis of progressive, liberal democracy inspired capitalism and socialism that combines the best of both worlds, an important question remains who should (co-)control vital resources and the means of production. In the Age of Abundance, we are all developing countries.
17. Perhaps counter-intuitively, this chapter advises to draw inspiration from the good parts of the Chinese innovation system; provided these elements correspond with our Western way of life (freedoms) and our participatory democracy. We should combine these ingredients with implementing the ancient institution of German regional development banking, which is responsible for the continued strength of German Mittelstand industries. It avoids the limitations of traditional banking while promoting quality, productivity, stability and economic growth. Even though China is a systemic rival of the US, and their ideology is incompatible with democracy, we must still be open to learn from Chinese poverty reduction by creating a knowledge economy, developing green, decarbonizing technologies, long-term planning in combination with decentralized experimentation, and more efficient, productive state control. We should transplant the well-functioning parts from the Chinese approach that are compatible with the human rights and freedoms we cherish, into our own democratic, post-scarcity systems. What's more, we should learn from history and consider implementing measures inspired by the social New Deal programs of the 1930s that helped the United States recover from the Great Depression, such as the Works Progress Administration (WPA), enacted by President Franklin D. Roosevelt.
18. This chapter views historic, contemporary, and future property paradigms as stages in growth of social responsibility. When addressing access vs. excludability dilemma's in a relative abundance setting, policy makers should not be afraid to experiment with different modalities of property, Roman law inspired multilayered property arrangements, common-pool resources (hybrid public-private goods), eliminating artificial scarcity, strengthening the public domain, Public Property from the Machine (=replicator), declaring/categorizing primary resources such as data as merit goods, and regulatory sandboxes. More specifically, the chapter considers both ancient and modern forms of common, collective and private property and proposes a socially equitable bundle of property rights tailored to the Age of Abundance. An ownership arrangement that connects property to liberty (and reward), and decouples it from status and respect, in particular from negative social recognition. In practice, decoupling property from status will be a quantum leap.
19. The chapter advocates for awareness of the mental, ethical, social and cultural shifts essential for change. It discusses post-materialist values fitting the post-scarcity economy, such as altruism, solidarity, and truth. Much work needs to be done in this area. These redefined values and ideals are operationalized in the Equal Relative Abundance (ERA) principle of distributive justice. Critically, post-scarcity values have to be actively embedded in our technology. Companies and the state have a mutual responsibility for the design, architecture and infrastructure of 4IR technologies. Impact assessments have to be employed. As society shapes technology, technology shapes society.
20. Given the evolutionary factor that human nature keeps striving for more (wants) even when its needs are fulfilled, the road ahead will not always be easy. Political conservatism, the implications of the theory of path dependence, and market power of incumbents that have an interest in status quo will obstruct a smooth transition. Negative sum games must be solved, positive sum games pursued. In this light, the chapter lists 15 barriers and 15 enablers of abundance.
21. The central thread through this chapter is the role of technology as an engine of change. Naturally, technology is not the prime cause for all our difficulties, nor is technology our only salvation. Having explored normative parallels between managing exponential technologies and abundance, the chapter concludes that the reforms necessary to balance the socio-economic effects of 4IR technology now, fit the trend of a shift from scarcity to well-managed relative sustainable abundance for all, on the planetary level. The proposed reforms address the identified challenges concerning the equal distribution of burdens and benefits across members of society. Thus, when policy makers execute the suggested 4IR reforms using good governance practices -being enablers of abundance-, they automatically make society ready for the post-scarcity economy. Addressing the identified systemic challenges requires cooperation on a global level.
22. The chapter ends with the utopian realistic prediction that during the Age of widespread relative Abundance, having mastered the art of good governance and equality, people will be free to spend their time on understanding the art of living, and on what it means to be human.

## Introduction

Over the past decades, exponential increases in productivity have resulted in dramatically lower manufacturing costs, while markets have spread well beyond national borders, resulting in larger economies of scale (Sadler, 2010, p. 46). Globalization, digitization and intensified competition made prices for a broad spectrum of products and services fall toward the marginal cost of production (Sadler, 2010). Ubiquitous technologies are eradicating scarcity in many industries. These macroscopic system trends are causing our economy to transition from relative scarcity to relative abundance.

Our market economy is not the only thing changed by the transition from scarcity to abundance. Conceptually, the economy is part of a larger system: society. In addition to the economy, this system consists of our socio-political institutions and our environment. In the words of Philip Sadler,

“*there are three distinct but interdependent systems—environmental, sociopolitical and economic—which continually interact to create, on a global scale, an all-encompassing system resulting from the complex feedback loops existing between the three sub-systems.”*

All 3 systems are under pressure due to the trends identified. Not just the economy, but society as a whole is undergoing a metamorphosis into the Age of Abundance. Hence, the technology driven post-scarcity society is upon us.

This chapter views the concept of society through a broad, interdisciplinary lens. On the one hand, it emphasizes the institutional, ordering aspects of society: the state and the state apparatus, as justifications for coercive power and political authority. On the other hand, it sees society as a community linked to a certain territory or geography, which share a common way of life, morality or purpose. It understands society as a complex system evolving from an individual level to a group level, as a collaborative framework designed to produce distinct outcomes such as wellbeing and prosperity, structured around a coordinated network of relationships between people and their environment, including their traditions and cultural identity. The essence of a society is the intrinsic desire/striving of people for survival, connection and social interaction. The concept of society has diverse appearances, configurations and dimensions.

While framing key aspects and goals of present-day societies and describing their shift to a state of pervasive relative abundance, we can draw historical timelines of progressing forms, or evolving types of society. After all, as 4IR technology is exponential, time is linear. In chronological order, these are Hunting-Gathering societies, Horticultural societies, Agrarian/Feudal societies, Industrial societies, Post-industrial societies, Information and Knowledge societies, and Abundance societies.

Society as a concept can be studied and defined from various scientific disciplines, such as (1) political science, as in the science concerned with the study of the establishment, conduct and effects of government policy; (2) sociologically, as in the study of the social behavior and social action of man in society; (3) anthropologically, as in the science concerned with the study of people and cultures in all their aspects; and (4) philosophically, as in the origin, meaning and essence of society.

The Abundance Society concept consolidates these notions, as much as scientifically sound. During the transition to the Age of Abundance,^1^ more and more forms of global governance will be put into operation, conceptually separating the abundance society from territoriality and from the nation state. And so, the abundance society evolves into a technologically advanced global human civilization.^2^ As a large, networked sphere in which Earth's regions and nations, and people's socio-cultural identities are united. In that sense, the Abundance Society is a macro model of a world system.^3^ This technology propelled post-scarcity model embeds economic, legal, ethical, socio-political and cultural anthropologic insights into an enduring cooperation of people having shared interests, common institutions, and collective minimum standards of living. In such a cosmopolitan abundance society there is ample room for divergent values (although post-materialistic values will become the [leitmotif] dominant theme, superseding social stratification) beliefs, identities, cultures and traditions, and opportunity for a plurality of worldviews (such as Eastern or Western) and beliefs, as long as these respect the overarching Equal Relative Abundance (ERA) paradigm, which will thus be the highest in rank.

Our starting point is that relative abundance is not utopian. Abundance is not a myth. Scarcity has a beginning and an end (Xenos, 1987). Technology will erase scarcity in more and more economic areas in the foreseeable future, but not everywhere or for everybody. Besides that, technological progress is often at odds with the law, in particular property law, antitrust law and IP. For many people in the world however—in both developed, developing, and underdeveloped countries—the notion of an Age of Abundance will sound utterly bizarre and totally misplaced. There is a tension between abundance and equality.

The transition to the post-scarcity economy, and at a higher level the abundance society, requires addressing a number of key points of interest, which we can categorize into the 3 parts of the all-encompassing system. For example, abundance is at odds with the functioning of the market and the conduct of financial institutions in a capitalist model, with social equality and poverty, and with sustainability and climate change. According to Lukas Peter, the 3 constituent systems are all in crisis. He speaks of “*the existing political, economic, and ecological crises that humanity faces*” (Peter, 2021). These problems demand reforms and system change. These reforms should prevent stagnation and decline, and incite progress. In this context, we can identify enablers and barriers that will facilitate and accelerate or, on the contrary, delay or prevent the transition to an Age of Abundance. Ultimately, our goal should be to mitigate inequality and achieve widespread abundance for all.

Anticipating these grand challenges, policymakers should acknowledge the tensions and modify and improve the functioning of our socio-economic, legal and political institutions, by employing clear goal-setting activities. To this end, good governance, according to democratic principles and high ethical standards, is key.

Parties with vested interests will vehemently oppose these changes and the associated reforms. Some will remain unaware of the changes. Others, who recognize the transition and aspire to steer it in the right direction, will come up with different solutions, depending on their beliefs and the information available to them. Good governance should manage all of this. The stakes are high. Managing the shift to the Age of Abundance could either lead to the end of our species, or to an Age of Enlightenment. And everything in between.

Good governance considers in what manner the state conducts public policy, manages public resources and promotes overall prosperity (de Graaf and van Asperen, 2018).^4^ This chapter connects good governance to the end of scarcity and links equality to abundance. It provides suggestions on how resources and the means of production can be effectively managed in an affluent, “Cornucopian” society (DeLong, 2000, p. 3), with the aim of equitable outcomes for the masses instead of desirable results for select groups. To this end, the chapter introduces the Equal Relative Abundance (ERA) Principle of Distributive Justice, which should guide/inform good governance decisions. Moreover, ERA can be the basis for (inspire) a new social contract between the state, individuals and companies in the Age of Abundance.

This chapter critically examines the normative justifications of our scarcity based legal institutions, such as property and intellectual property (IP) systems, in light of the Fourth Industrial Revolution (4IR)^5^ and the post-scarcity economy.

The chapter seeks to provide concrete solutions for the identified multidimensional challenges, focusing on the concept of scarcity in economics, law and sociology, entrepreneurial conduct, consumer behavior, cultural norms and post-material values, socialized property paradigms, regulatory frameworks, and forward-thinking policy interventions. In addition, it offers philosophical viewpoints on augmented socio-economic systems that make sense in post-scarcity conditions, such as democratic post-capitalism.

An Age of Abundance requires a government system tuned for abundance. When thinking through such a system, we need to reconcile social, economic and political theory, considering the function and purpose of the state. The chapter puts the emphasis on good governance in the sense of well-managed relative sustainable abundance, in concert with the individual responsibility and choices of the various stakeholders themselves, including citizens and companies, that together form society and each have a share in the way in which it is shaped.

The central thread through this chapter is the role of technology as an engine of change.

## The concept of scarcity in economic theory

In economic theory, the concept of scarcity is understood as the difference between finite resources and infinite wants (Samuelson, 1980). Scarcity refers to the gap between limited commodities in the form of supplies and theoretically unlimited needs in the form of demands by the market, the state or the commons. Scarcity has an impact on the economic value consumers place on goods and services traded on the marketplace, as well as how governments and private businesses allocate resources.

Economic scarcity's causes can be categorized into three types: demand-induced, supply-induced, and structural (PRB, n.d.). Scarcity pertaining to resources that are limited in quantity can be relative or absolute (Raiklin and Uyar, 1996; Baumgärtner et al., 2006; Daoud, 2011, p. 41).

According to Daoud, relative and absolute scarcity

*refer not only to different objects (physical vs. social), different states (post-scarcity), or different spatial positionings of resources (extrinsic vs. intrinsic), but actualsly to different kinds of scarcities* (Daoud, 2011, p. 41).

Contemporary economics denies the possibility of abundance (Dugger and Peach, 2009). In other words, current economic theory only finds value in scarce commodities. That is problematic, given the fact that most first world countries find themselves in the midst of a change from a post-industrial economy to a post-scarcity economy. Put differently, the economics of how products and services are created and distributed change when scarcity is removed from the equation. This means that we have to design a different kind of economics: one that addresses relative abundance.

In the words of Dugger and Peach,

“*The modern world needs an economics based on modern notions of widespread abundance and equality rather than concepts of scarcity and inequality.”* (Dugger and Peach, 2009)

The same applies to society's social and cultural institutions, such as political, government, economy, legal, business, finance, education, healthcare and work systems. Our institutions were built on the basis of preindustrial scarcity economics and must now evolve into institutions based on abundance economics. Whereas, economics should be redefined, so do our institutions.

## Relative scarcity and relative abundance

Scarcity is the antipode of abundance.^6^ As diagnosed above, it is important to keep in mind that scarcity and abundance are (in most cases) relative concepts. In economic terms, almost everything is scarce or abundant to a certain degree, such as physical goods and digital services. Examples of absolute scarcity and absolute abundance are in fact rare. Money and natural resources such as water, sunlight, air and even human intelligence and creativity: usually these are moderately scarce (Tebble, 2020). With time being the exception to the rule, as this is an absolute scarcity in the sense that it is limited by nature, but not in relation to demand.^7^ Supply of time is naturally limited and we can do nothing to increase supply. Another economic distinction we can make is that between finite and infinite goods. Or between scarce and unscarce goods.

Artificial scarcity, on the contrary, refers to the purposeful limitation of supplies, products, services and access to information despite the fact that the technology and production capacity, as well as the ability to share, exist to generate an abundance (Hai-Jew, 2020). The goal of creating artificial scarcity is typically to raise either prices or demand. Examples of artificially constructed scarcity are intellectual property (IP) such as copyright and patent, monopolies, technological protection measures such as paywalls (Sullivan, 2016), and NFT's (non-fungible tokens) (Artificial Scarcity - Wikipedia, n.d.).

In addition, we can interpret the concept of abundance as having (adequate or sufficient) levels of primary and secondary necessities at hand at zero-marginal cost: the necessities of life such as food, water, shelter, and healthcare, as well as education, recreation, self-expression, transportation, and personal security (Dugger and Peach, 2009).

Rafikov and Akhmetova advocate a wider lens, even disconnecting scarcity and abundance from its economic dimension, as the idea of traditional economics only leads to competition in the negative sense of the word: to confrontation and conflict, instead of kindness, empathy, cooperation and sharing. They argue that:

*simplicity, spirituality and universal values are necessary to remedy the ills of overconsumption/overproduction, waste and inequality* (Rafikov and Akhmetova, 2019).

This chapter views relative scarcity and relative abundance as temporal socio-economic categories at two opposite sides of a continuum. This means that a shift from scarcity to abundance can take place, when certain subjective and objective circumstances or criteria have been met pertaining to the evolution and emancipation of the social and political order, which should be sustainable, (and the environment), beyond the notion of basic material needs (Giddens, 1996). This evolution will be driven by a marriage of technological progress and human choices.

What is—subjectively—perceived as scarcity by one person, can be more than sufficient for another. What's more, we have to deal with the evolutionary factor that human nature keeps striving for more (wants) even when its needs are fulfilled (Keynes, 1963; Raiklin and Uyar, 1996; Baumgärtner et al., 2006; Daoud, 2011, p. 41).^8^ Our current economic system is perfectly suited for a constant push for growth: capitalism.^9^ In an objective sense, the presence of adequate or sufficient levels of relative abundance with regard to primary and secondary necessities of life is related to the degree of technological development and socio-cultural evolution^10^ of a particular society (Dugger and Peach, 2009, p. ix–x).^11^ Therefore, relative scarcity and relative abundance are related to human behavior and to the design of our socio-economic, legal and political institutions.

The constant pursuit of growth and progress does not seem problematic in itself, as long as it is keeping pace with technological and socio-cultural advancements.^12^ Put differently, the blind chase of growth based on materialistic values is problematic, the moment it causes disproportionate damage to the 3 analytical components of our all-compassing system (Sadler, 2010, p. 234).^13^

This makes clear the importance of conducing interdisciplinary research on the relationship between economy, society and environment, against the background of integrated concepts such as scarcity, abundance, and sufficiency (SAS) (Daoud, 2011, p. 1, 42).^14^ The precise character and nature of scarcity, abundance, and sufficiency is crucial, as is their interplay. Greed, conspicuous consumption (Theory of the Leisure Class: Veblen, Thorstein, 1857-1929: Free Download, Borrow, and Streaming: Internet Archive, n.d.)^15^ and winner takes all effects must be addressed, aiming for a more balanced distribution of the realized relative abundance—or sufficiency—over the world population.^16^ This involves a culture change, preferably within 1 or 2 generations. Relative abundance ought to be well-managed by government, market, and people in concert, with clear rules about their mutual relationship. It should be managed in a way that preserves the ecology of the earth and its surrounding universe.^17^ An institutionally balanced trias politica (Smismans, 2002) based democratic socio-political system should coordinate this, with the state divided into three organs that monitor each other's proper functioning.^18^

The goal for humanity should be to strive for equally distributed, relative sustainable abundance.

## The end of scarcity

The end of scarcity has been described from many perspectives and schools of thought. The view that economic abundance is possible, and that it is a situation or condition that we as an economy and society want to move toward is widely shared (Dugger and Peach, 2009). Opinions differ on how to get there, and how it will look like. The diagnoses of challenges and solutions offered often run along the lines of ideological preferences.

This can be illustrated by a short selection of highlights from the history of abundance.

In 1798, Malthus warned of the link between abundance and overpopulation (An Essay on the Principle of Population, as it Affects the Future Improvement of Society. With Remarks on the Speculations of Mr. Godwin, M. Condorcet and Other Writers: [Malthus, T. R. (Thomas Robert), 1766-1834]: Free Download, Borrow, and Streaming: Internet Archive, n.d.). In this regard, Sadler sees overpopulation as part of the problem (Sadler, 2010), whereas Dugger and Peach consider population growth in combination with 0% unemployment or 100% labor force participation as a necessary solution to achieve optimal production levels wanted for the transition to a state of abundance, arguing that abundance itself may be a form of population control (Dugger and Peach, 2009).

Mill too foresaw the end of scarcity in 1848 (Mill, 1976, p. 260), his north star being the steady state: an ultimate goal characterized by a stationary equilibrium between population and capital (Boulding et al., 1978). To reach it, Mill assumed abundance enabling conditions such as long-term peace, law and order, and full employment connected to optimal levels of productivity (Xenos, 1987; Gallarotti, 2000). According to Heilbroner, Mill

“*prophesied the transformation of capitalism, in an environment of abundance, into a balanced economy, in which the capitalist, both as the generator of change and as the main claimant on the surplus generated by change, would in fact undergo a painless euthanasia”* (Heilbroner, 1970, p. 282; Chernomas, 1984)

In the same year, Marx, the father of socialism, rejected capitalism and its scarcity postulate. Still, Marx strongly believed that a technology driven economy of abundance was possible, under the condition of “democratizing” the means of production, and the equal distribution of wealth (Marx, 1988). In addition, Marx points to post-materialism. In the words of Stillman,

“*since all needs be pursued, human beings reflect which needs satisfy; this cannot must to try to on reflection atomistic, leads, Hegel and Marx think, with the material and but to not concern with cultural and social needs and their satisfaction.”* (Stillman, 1983)

Keynes was the first to suggest the possibility of a less than full-employment capitalist equilibrium (Dugger and Peach, 2009, p. 13). For him, technical progress and capital accumulation, will eventually lead to a state of abundance, making capitalism and its acquisitive pathological values and preoccupations redundant (Keynes, 1963, p. 329; Chernomas, 1984). In 1928, in a post-materialist manner, he points to the real values of life, and the art of life that should be pursued once the economic problem has been solved (Keynes, 1963, p. 373).

In 1942, conservative thinker Schumpeter predicted the end of poverty, driven by a market free from government intervention, entrepreneurship, sparked by temporal monopolies, and technology propelled innovation, while emphasizing the sociological and political factors in society (Schumpeter, 1950, p. 66; Dugger and Peach, 2009, p. 11). However, the shift to relative abundance implicates creative destruction on such a scale that will involve complete industries to disappear, once prosperous regions becoming deprived, as new elites shall emerge (Sadler, 2010, p. 3, 42, 43). Thus, according to Schumpeter, growth spurred by creative destruction results in more equality in the sense of better living standards for everyone, but it will not be absolute, nor equilibrious, as profusion will not be everywhere and for everybody at all times. Put differently, there will always be a few big winners in a Schumpeterian economy, while society benefits as a whole.

Based on empirical evidence, Inglehart argued in 1977 that industrialized countries are moving away from materialistic values necessary to satisfy basic survival needs, toward post-materialistic values emphasizing autonomy and self-expression (Inglehart, 1977; Giddens, 1996). The Inglehart-thesis can be measured by determining the spread of post-materialism in a certain region or population group, such as young, old, gender, religion or income-based. Post-materialism can have a detrimental effect on economic growth though, which is debatably needed to reach widespread conditions of post-scarcity (Kafka and Kostis, 2021). Hence, although there seems to be a tension (paradox) between post-materialism, economic growth and the transition to the post-scarcity economy, post-materialist values are widely believed to be a social indicator of the dawn of an Age of Abundance.

Although unfavorable toward the idea of universalizing abundance in a Marxist sense, and the chances of building a social order that would support it, Giddens (1990) formulated his own conception of the possibility of a post-scarcity society (Giddens, 1995). Within an era dubbed post-modernity, he can see the contours of a post-scarcity system, coordinated on a global level (Giddens, 1990, p. 165). Instead of a distinct form of social order, Giddens views the post-scarcity society as a series of trends, surrounding life politics, manufactured risks, a decline in productivism, and the impact and value of technological innovation (Giddens, 1995, p. 8). Such a society would involve significant alterations in modes of social life, and a global redistribution of wealth would be called for (Giddens, 1990, p. 166). Giddens connects post-scarcity to equality, inclusivity and post-materialism.

Published in 1999, Rawls liberalism inspired Theory of Justice links the concept of moderate scarcity to distributive justice (Rawls, 1999, p. 109, 110; Xenos, 1987). Rawls followed Hume in postulating that, although it would be possible to justify certain exclusive property rights in a Cornucopian society, these rights would be unnecessary (Hume, 1995, p. 145; Tebble, 2020, p. 5, 6). In contrast, a society under conditions of scarcity should find a legitimate authoritative basis for the apportionment of scarce goods, in the form of laws and liberal institutions (Xenos, 1987, p. 237, 239).

Frase sketches 4 possible post-capitalist futures along the axes of two logical opposites: resource abundance vs. scarcity and egalitarianism vs. hierarchy (Jacobin, n.d.). Illuminating these foundational elements of a particular social order, he explores 4 simplified portraits of utopian and dystopian scenario's (located at the extremes of the post-capitalist spectrum): communism, rentism, socialism and exterminism. Using libertarian logic, the author rejects intellectual property as being imposed artificial scarcity, which is irrational, dysfunctional and barbaric even, especially in the digital domain (Boldrin and Levine, 2008; Jacobin, n.d.; P2P Foundation, n.d.).

In 2014, Rifkin (also) predicts the end of the capitalist era and the awakening of a new global collaborative commons (Rifkin, 2014). Fierce competition sparks revolutionary technological innovations such as new energy paradigms and the Internet of Things, that boost productivity to the point where the marginal cost of production approach zero, making products and services in essence free, and abundant, free from market forces (Rifkin, 2014). These trends result in decentralized production movements based on the economics of abundance, such as open source software, and institutions such as property ownership becoming increasingly redundant (Rifkin, 2014; Goodreads, n.d.). The shift from market capitalism to collaborative commons in a decentralized society, the author believes, shall bring about a change in values from exclusive ownership centered, to a mentality of sharing.

As such, there appears to be broad consensus among pioneers of the post-scarcity paradigm that a mindset shift toward post-materialist values is required throughout the transition to relative abundance. And that during the Age of Abundance itself there will be more time and opportunity for self-fulfillment, creativity and spirituality, after the materialistic wants en needs have been satisfied.

## Forecasting abundance

Anno 2022, certain goods are scarcer than others. The course from scarcity to abundance happens faster for digital and intangible goods and services such as books, music, film, information and knowledge, than for physical goods such as household goods, electronics, clothing, as well as services such as air travel (Sadler, 2010, p. 46). Besides this, the levels of relative scarcity and abundance are rather unevenly distributed over the world's population, resulting in rich and poor regions. As noted before, technology has eliminated, and will continue to undo scarcity in an increasing number of countries and economic fields, although not everywhere or for everyone. Naturally, developing countries are generally less technologically developed. The trend of unequally disseminated, technology driven relative abundance is one of the causes for a wealthier northern hemisphere, and a poverty-stricken, needier global south.^19^ This phenomenon of scarcity within abundance—be it on a local, regional or global level—is known as the post-scarcity paradox and is caused by the poverty trap/penalty.^20^

The 10 poorest countries in the world as of now are Madagascar, Chad, Liberia, Malawi, Mozambique, Democratic Republic of the Congo (DRC), Central African Republic, Somalia, South Sudan and Burundi (mostly former French colonies). To people living in these countries, the notion of the advent of an abundance society operating under the Equal Relative Abundance Principle of distributive justice must sound bizarre and utterly misplaced. Relative abundance seems far out of reach, even for sectors and industries in which it has been the most easily obtained by Western countries. These ideas will sound like a distant dream at best.

Some futurists hypothesize that the period of relative abundance is just behind us rather than society being in the midst of the transition phase to post-scarcity. According to them, we can expect another period of relative scarcity caused by rising population and resource depletion, before the world will ultimately experience an age of abundance (Aguilar-Millan et al., 2010).

Trends that seem to speak against the transition to relative abundance are the Russia-Ukraine war of 2022, the supply chain crisis of 2021 including the computer chips scarcity, inflation and rising prices, the energy crisis, a shortage of raw materials, scarcity or surplus of labor—or its sub-optimal distribution among the various professions. Are they natural fluctuations in demand and supply that follow an explainable pattern? Or is it coincidence? Are they trivial shortages and will we also have to take future scarcities into account during abundance, for example due to a pandemic, world war, volcanic eruption or comet impact? Time will tell whether these are transitional perils associated with the passage to a post-scarcity economy, counter-intuitive characteristics of the Age of Abundance, characteristics of the scarcity paradox, or barriers that delay the shift.

How will we know if we are really moving toward an Age of Abundance? Can we perhaps measure or predict it using quantitative methods? Relative abundance per sector or territory is not easy to measure using empirical methods, on which evidence-based policy could subsequently be developed. In the words of Boulding et al. (1978):

“*on the one hand, scarcity and abundance of certain prime resources have exerted a profound shaping influence on the evolution of the American sociopolitical system; on the other hand, the nature, extent, and consequences of different amounts—of the supply of any set of resources on hand as it were—is also a matter of conflicting perceptions and judgments and not necessarily of objectively determined actuality.”* (Boulding et al., 1978)

Forecasting abundance can seem like an impossible task due to the many variables involved, similar to forecasting the weather.

An interesting way to predict the degree of technology driven relative abundance, is technology forecasting. Given that we assume that the transition from relative scarcity to relative abundance is to a large extent technology driven, it makes sense to connect the technology forecasting method to forecasting abundance. Applying this method can give us more control over the timeline, the expected developments, and the necessary/obvious policy strategies.

According to Huang et al., tracing historical progression and forecasting future trends of technology evolution is essential for government science and technology planning, and formulating coherent enterprises R&D strategies and policies (Huang et al., 2017, p. 185). Instead of focusing on single factors, monitoring the patent landscape and the technology maturing process through applying systematic co-classification, co-word and main path analysis of patent citations *via* machine learning techniques, can help revealing the technical evolution process of a certain technical field, such as 3D printing, AI or quantum computing (Huang et al., 2017). This quantitative method allows us to detect previously unknown patterns, and discover significant clues about technology hotspots and development prospects (Moehrle and Caferoglu, 2019).^21^ Outlining technology evolution pathways are essential to track innovation progress, and can assist decision-makers in guiding technology development and formulating plausible, evidence based innovation policies (Moehrle and Caferoglu, 2019).

As stated by Zhang et al. (2019), conceptual and empirical investigation of technological convergence is the key to understanding indicators and drivers of technological emergence in its varied dimensions. The authors

“*approach ‘technical emergence' from a broad perspective of science, technology, & innovation (ST&I)—e.g., advances in scientific development and in technical evolution, as well as in emergent commercial innovations. Understanding processes of technical emergence becomes essential for technological forecasting investigations at either macro or micro levels—e.g., technology roadmapping, technology delivery system modelling, approaches to identify drivers of technical emergence, and other perspectives such as empirical assessment to validate prior forecasts.”* (Moehrle and Caferoglu, 2019)

With technological convergence, I mean the process—or phenomenon—by which originally independent operating information technologies are growing together or integrate to form new synergies (Papadakis and Lovitt, 1977).

Science:

Researchers should unite theoretical and quantitative disciplines of economy, law, political science, philosophy of science, ethics, psychology, biology, anthropology, and history, in order to devise responsible and sustainable scientific solutions for the problems identified above. These solutions should be debated in multidisciplinary, inclusive teams of scholars stepping outside of their research silo's, with the

“*capacity and willingness to transcend the constraints of specialization.”*^22^

We conduct these studies in a structured and categorized way, per part of the all-compassing system (sector or issue-specific), but also from a holistic, macroscopic post-scarcity helicopter view.

Policy:

What is needed on the policy front right now, is a combination of lateral thinking, evidence-based strategy making, and utopian realism. Translated to 2022, Giddens teaches us that—in order to survive modernity—we have to create policy models that balance utopian ideals with realism, which address the potentially existential threats to humanity posed by exponential technologies, with the end goal of a good society that is liberated from consumerism (Theory of the Leisure Class: Veblen, Thorstein, 1857-1929: Free Download, Borrow, and Streaming: Internet Archive, n.d.), inequality and servitude (Giddens, 1990).

Applying Heidegger's philosophy, we would add that it is vital for the survival of humanity to have a relationship with technology based on freedom and truth (Heidegger and Lovitt, 1977). Humans—including entrepreneurs, consumers, research institutions and the government—must urgently pursue and shape our relationship with technology in accordance with commonly shared democratic principles, based on mutual values of freedom and trust. We should actively build these principles and values into our technological systems and infrastructure as much as technically achievable, from the first line of code.

## Technologies of abundance

Exponential innovation fuels the transition from relative scarcity to relative abundance. High-velocity (speed) innovation is driven by transformative, exponential technology. Exponential technologies enable abundance. Over the past decades, a number of disruptive, ground-breaking 4IR technologies have been rapidly propelling and shaping the transition to a post-scarcity model (Huvila, 2012, p. 35). These are artificial intelligence and big data, the family of quantum technologies, nano-technology, biotechnology, 3D printing, nuclear fusion, distributed ledger technology (DLT), virtual reality, and hyper-accurate positioning, including technological synergies and hybrids. Each have significant social and economic impact, and the potential to increase living standards (Sadler, 2010).

It is important to realize that these are all exponential technologies, in the sense that their evolution is not linear, incremental and materializing according to Darwinian patterns, but at an exponential rate.^23^ This means that their social impact, once diffused and absorbed, will become ever greater and more radical, causing the transition to widespread post-scarcity conditions to take place faster and faster.

An interesting feature of exponential technology is democratization. Technology permits more and more democratization of innovation (von Hippel, 2016). Democratization, not in the sense of expropriation but in the sense of, for example, facilitating machine learning and quantum computing power *via* the cloud, makes technology omnipresent. The combined use of pervasive digital technologies and web 2.0 services has layered, amplified network effects on progress and growth, and enables completely new business models driven by low productions costs and free pricing structures (Sadler, 2010, p. 29). Because of these parallel, synergistic multiplier effects, developmental progress, close to zero cost reductions, and societal change happen at an increasingly accelerated rate. With that, exponential technologies facilitate the shift toward a state of relative abundance in an increasing number of industries, economic sectors and even complete societies.

Naturally, technology is not the prime cause for all our difficulties, nor is technology our only salvation (Boulding et al., 1978).

### Abundance enabling technology policies

But because their implications are so far-reaching, we have to look closely at the features and design of these technologies. As society shapes technology, technology shapes society. That is why our values must be proactively embedded—i.e., ex ante—in the design of our technology, before it is diffused into society. An example of ethically aligned design are the alignment techniques as applied in InstructGPT.^24^

In addition, life cycle auditing of agreed upon legal-ethical values is required. These values should fit within the society we have in mind, emphasizing solidarity, altruism, post-materialism, freedom, autonomy, democracy, and truth. The manner in which these values are operationalized will be contextual and dynamic, as society is in constant flux. Policy makers must introduce adequate laws and policies ensuring universal, core ideals, standards, values and institutions are integrated. By extension, the technologies' features should contribute positively to the shift to the Age of Abundance. Principles of good governance require this.

Below I list a catalog of 10 technologies of abundance. Besides giving a definition (1) and stating the reason why they are exponential (2) and thereby remove scarcity, I provide recommendations (3) that will enable the shift to an Abundance society in which our 4 identified main problems are sufficiently solved/addressed, and any transitional barriers will be removed. Hence, we think in terms of enabling abundance and removing roadblocks. The law and policy below suggestions should be viewed in conjunction with each other—mainly because of synergies, hybrids and technological convergence—and can be applied to adjacent fields as well. This applies in particular to universal, core horizontal rules, which can, e.g., be applied to both AI, nanotechnology and quantum technology. Vertical, sector specific rules will be more special, and different, in line with existing quality management systems (QMS) per industry (Kop, 2021a, p. 435).

#### Artificial intelligence

1. Artificial intelligence (AI) can be defined as either an entity, a system or a science (Emerj Artificial Intelligence Research, n.d.). AI can be described as an intelligent machine that can think and plan strategically. As an agent possessing cognitive functions and skills usually associated with humans, such as learning and reasoning (Kop, 2020a). Today's AI systems have various components, such as machine learning algorithms, recursive neural networks and the inference system. A neural network is an artificial, mechanical emulation of the connections between nerve cells existing in the human brain.
  A powerful example of synergistic effects between technologies are living, organic robots that can procreate using a combination of molecular biology and artificial intelligence. These AI-generated xenobots have the ability to reproduce using kinetic reproduction.^25^
2. AI is the textbook example of an exponential technology that fosters relative abundance. Ubiquitous AI is expected to be more transformative than the societal impact of electrification (Sadler, 2010).
3. Building on the ancient Roman multi-layered property paradigm (Rahmatian, 2011), a new model of AI specific non-exclusive propertization can be imagined that burdens no one—at least no legal person—and benefits all. To this end, lawmakers should introduce the legal category of res publicae ex machina (public property from the machine), which results in AI inventions and creations that have no human input in the chain qualified as public domain subject matter, free to use as the air we breathe around us (Kop, 2020a, p. 326–328). In other words, non-IP or exclusive property can be vested in these AI generated creations and inventions. Res publicae ex machina can be compared to the output of the Star Trek replicator,^26^ and should be confirmed by a formal, government issued PD stamp until people are so accustomed to an AI's output not being converted into private property that this public domain notion will become part of our legal culture, including opinio iuris sive necessitatis.^27^ An enriched public domain increases relative abundance conditions.

Please note that the legal category of public property from the machine, or even declaring AI a public good, will be easier to justify in an abundance society. We will see in paragraph 7 that as relative abundance conditions become more widespread, there will come a moment that IP will lose its legitimacy and justifying IP as an instrument of artificial scarcity becomes harder.

#### Big data

1. Big data refers to the exponential amount, velocity and diversity of contemporary datasets. Data has become a primary resource for both humans and machines (Kop, 2021d).

Data science is a young, emerging interdisciplinary field that includes focus areas including mathematics, statistics, econometrics, artificial intelligence, blockchain, algorithms, business analysis, and pattern recognition. It integrates concepts from economics, business administration, law and ethics. Data science is thus dominated by views originating from the alpha, beta and gamma sciences.

2. In smart cities and the Internet of Things, the amount and complexity of AI input and output data is growing exponentially under the influence of data generating devices, the cloud and 5G network technology. The amount of data exchange is massive. In this segment, we focus on the machine learning training, testing and validation datasets, or AI input data. Thus, big data can be labeled an exponential technology that facilitates relative abundance.
3. Because AI needs big data to develop, it is important to remove barriers to access, sharing and use of this so-called AI “input” data. Practical solutions to achieve these goals, are concepts such as fair learning (Lemley and Casey, 2020), a novel right to process data based on a de lege ferenda quasi usufruct (a *ius utendi (usus) et fruendi (fructus) without a ius abutendi (i.e.*, no *pars dominium* or *proprietas)*, not for land, but for data) (Kop, 2021d), and data altruism in the form of data donorship (Kop, 2021d). This pertains to all taxonomies of data, such as government data, R&D data, personal data, commercial data, and mixed datasets (OECD, 2019).

The timing for these reforms is rights, since, at the moment, a clear legal basis for the primary and secondary use of input data for machine learning purposes is missing in the US (Kop, 2021d). This loophole leads to legal uncertainty and to costly lost opportunities from less powerful or less well-trained/developed AI systems, which are—if done right—important enablers of abundance.

#### Quantum technology

1. Quantum technology originates from applied principles of quantum mechanics (superposition, entanglement and tunneling), the theory of the very small (Peebles, 1992). Quantum mechanics attempts to explain the interaction between matter and energy and the building blocks of atoms at the subatomic level, beyond classical physics as described by Einstein's general relativity, the theory of the very large (Einstein, 1905). The family of quantum technologies has various application areas. We can distinguish quantum computing, quantum communication including the quantum internet, quantum sensing and metrology, quantum simulation, fundamental quantum science, and artificial intelligence.
2. Real-world quantum driven systems, products and services are expected to have far-reaching socio-economic impact (Kop, 2021f). Synergies of quantum computational paradigms and AI are believed to generate an intelligence explosion, and provide the world with a new perspective on science itself (Kop, 2021c). With that, the family of quantum technologies has all the features of an abundance enabling technology.
3. Introducing legal-ethical frameworks for quantum technology (Kop, 2021f), accompanied by best practices and codes of conduct in the form of quantum impact assessments will result in awareness of the ELSPI implications of this promising technology (Kop, 2021g). I recommend a risk based regulatory approach that focuses on avoiding apocalyptic scenarios per quantum application and per industry, such as cryptology, chemistry, energy, defense, and finance. In addition, our innovation architecture should be constructed, so that benefits will be distributed equitably and risks proportionally addressed, without stifling innovation (Kop and Brongersma, 2021).^28^ The latter for instance by introducing legal sandboxes that afford breathing room to develop and test experimental quantum technology, when certain safety requirements have been met.

Governance of the imminent quantum internet, which uses quantum physical phenomena and quantum network and communications technology, we build upon experience gained and lessons learned from managing the internet as we know it today, including addressing risks, sustainable commercialization and maximizing the social value of shared infrastructures (Greenstein, 2015).

#### Nanotechnology

1. One step up on the micro scale, nanotechnologies are also technologies of the very small. Nano-systems, devices and materials refer to the various methods, configurations and designs pertaining to the molecular level between 1 and 100 nm. This includes molecular manufacturing.
2. Nanotechnology is foundational for AI enabling hardware, due to the large-scale integration of semiconductor nano-transistors into computer chips, 3D integrated circuits, graphene computing, and the use of photonics in optical computing (Brongersma, 2021). Further, nanotechnology enables mass DNA sequencing at affordable costs, is able to manufacture extremely durable materials, and can enhance living conditions through the ethical use of health-wearables and body implants. At the nanoscale, quantum effects become unavoidable. With that, nanotechnology can be qualified as an abundance enabling technology.
3. Nanotechnology can be used for good and for bad. As AI and quantum, it is classic dual use technology. These features of nanotechnology demand for enforceable dual use legislation, which includes licensing schemes and export controls (European Commission, n.d.). In addition, environmental legislation should proactively deal with the impact that tiny nano particles have on our planet (Sadler, 2010, p. 80). These measures should ensure that the societal benefits of nanotechnology outweigh its harms.

#### Biotechnology

1. Biotechnology is the technological application of biological knowledge, and involves the use of living systems and organisms, animate and inanimate materials, to develop products and systems. The applications of biotechnology in science and engineering, and industries such as pharma (Medication) and agriculture (GMOs) are countless. From heritable genome and gene editing, through CRISPR CAS designer baby's, to synthetic cells and biological computers.
2. Biological computers, or biocomputers, arise from a fusion of AI, nanotechnology and biotechnology. They partly consist of naturally occurring components. These machines, techno-optimists believe, will be able to self-improve by rewriting their own DNA (Sadler, 2010, p.74). Similar to quantum computers, biocomputers do their calculations by means of parallel computing, and not on the basis of the classical binary, serial computer system. Calculations are performed synchronously, rather than asynchronously. Biocomputers use a nanoscale fabricated network which provides directions for many protein filaments (actins) traveling simultaneously (parallel computing) through it. Powered by tiny molecular motors (myosins) that convert chemical energy into mechanical energy. The myosin guides the actin accurately through the channels of the artificial nano-network. The solution in the network corresponds with the answer to the mathematical question posed *via* the biocomputer. These calculations have been shown to be correct. An important advantage is that existing algorithms—after being optimized—can be used on biocomputers.
  Considering these facts, biotechnology including synthetic biology and synergies with other neighboring technical fields is an abundance enabling technology.
3. As patent law is technologically neutral in theory, but technology specific in application, anticommons concerns in biotechnology could demand patent reforms (Burk and Lemley, 2005).^29^ Specifically, these reforms should tackle wasteful underuse of patented biotechnology caused by fragmented exclusionary rights, after such innovation distorting underuse has been confirmed by replicated empirical research. In addition, bioethical principles should be incorporated in law, as law and ethics go hand in hand, and ethics alone can never be enough to regulate a specific technical field (Häyry, 2017).

#### 3D printing

1. 3D printing is a general purpose technology for design and production, that can be characterized as a laser printer capable of creating virtually any three dimensional physical object based on digital design, from fibers, polymers and fabrics, to organic materials such as living cells (bioprinting) (Desai and Magliocca, 2014). A personal factory that affects various levels and environments of manufacturing: at home, startup, scientific and industrial (Desai and Magliocca, 2014).
2. 3D printing has many benefits, such as office-based rapid prototyping huang (Huang et al., 2017, p. 11). It may give rise to a revolution in production, inspire creativity, and provide solutions to environmental challenges (Huang et al., 2017). 3D printing effectively democratizes and decentralizes innovation (von Hippel, 2018). The combined use of 3D printing, robotics, synthetic biology, AI and nanotechnology, powered by nuclear fusion, could lead to the realization of a machine comparable to Star Trek's replicator (Lemley, 2015b; Beebe, 2019). The only thing needed then, would be raw materials as input. Star Trek's replicator has been widely associated with abundance (The Economics of Star Trek. The Proto-Post Scarcity Economy | by Rick Webb | Medium, n.d.; The New York Times, n.d.).
3. Antitrust and consumer friendly laws should facilitate the transition from the legacy economy to abundance conditions. Incumbent firms, such as the ones whose market share comes under pressure due to 3D Printing, might have incentives to delay or prevent this transition *via* political rent-seeking (Mehra, 2016). In addition to revitalizing antitrust regulation, copyright and patent law should foster progress instead of artificially constructed scarcity (Desai and Magliocca, 2014; Lemley, 2015a).^30^

#### Nuclear fusion

1. Nuclear fusion is the universe's choice for how it generates energy (Massachusetts Institute of Technology, n.d.). Fusion energy is the process that powers the sun. Instead of nuclear fission used by existing nuclear power plants, nuclear fusion produces energy by merging hydrogen atoms (Ball and Thompson, 2021). In contrast to nuclear fission, it is clean: no radioactive waste. Scientists are getting better and better in capturing and scaling this energy source using various methods, such as magnets and fusion plasma reactors.
2. A scalable star in a bottle is a major step toward a decarbonized society. Climate change is a major barrier to widespread abundance for all. According to contemporary science, decarbonization can mitigate the negative effects of climate change. In addition, once the energy transition has been completed, energy scarcity will be a less frequent cause of global conflict. With that, nuclear fusion can be characterized as an abundance enabling technology.
3. An effective instrument that would make companies that are causing damage to the environment is internalizing negative externalities for competing harmful technologies like oil, coal, *via* taxes (Lemley, 2005; Abbott, 2020). That public money should be spent on clean energy sources like nuclear fusion and green hydrogen (Dugger and Peach, 2009; Rifkin, 2014). Moreover, controlling the supply and production chain, from mining lithium and cobalt to manufacturing to consumption to recycling, is a matter of geopolitics, that requires changes on a global level. It is expected that after the energy transition to a decarbonized society has been completed in 2050, things will calm down geopolitically because there is no longer a need to fight over scarce things such as oil, lithium and cobalt.

#### DLT/Blockchain

1. Blockchain is a form of distributed ledger technology. Blockchain is a decentralized registry of transactions connected by a peer-to-peer network. This system is based on distributed ledger technology (DLT). We distinguish between public and closed blockchain networks. This ledger is essentially a distributed database, with general participant agreement about the additions made in chronological order. The Ethereum platform is a form of a horizontal, permissionless network where all users have the same rights.

Blockchain is the revolutionary technology known from trading cryptocurrencies such as Bitcoin, Ethereum, Litecoin and various altcoins. Blockchain technology is just as groundbreaking as the invention of TCP/IP protocols. Cryptocurrencies are virtual, digital coins that are traded online and allow consumers and businesses to pay for goods and services. Bitcoins can be mined—often in large computer/server farms. Virtual coins are kept in an online or offline wallet, such as Ledger or Trezor, which are safeplaces for cryptocurrencies. Each crypto coin has its own apps to manage, buy and sell them. Trading in cryptocurrencies is often done through Bitcoin Brokers and Trading Sites.

The latest form of DLT are non-fungible tokens (NFT's). NFT's associate IP assets with a cryptographic token enshrined in a digital ledger, and create artificial, constructed scarcity (Fairfield, 2022; Zahr, 2022).

But besides crypto and NFT's, DLT can do many more things. Blockchain and smart contracts are suitable for the registration of property rights in material and intangible objects such as land, jewelry, containers or musical works. As of recently, blockchain apps have been available in the construction world, domotoca (e.g., the self-conscious blockchain house with automated maintenance decision making) (Wearetheledger, n.d.), and are applied in the food industry, the shipping sector, the jewelry industry, as an escrow agreement, for cloud computing, as a bank guarantee, in public transport and in the agricultural sector. Moreover, the decentralized nature of blockchain is a real alternative to the traditional top-down structure of companies. DLT promises less hierarchy, and more equality.

2. Artificial intelligence and blockchain can complement and reinforce each other: synergies of AI and DLT have the potential to solve the AI blackbox problem, as blockchain can be useful in analyzing the output of artificial intelligence. Think of opening the AI Black Box and explaining decisions, predictions and inferences. Transparency is a privacy enhancing technique and results in security by design, fostering sustainable innovation and enhancing trust in 4IR technology by the general public. For these reasons, blockchain/DLT is a relative abundance enabling technology.
3. The energy demand of DLT, and in particular of cryptocurrencies, is problematic and unsustainable. The energy consumption we are witnessing in bitcoin farms is far from environmentally friendly. In addition, the rate of these currencies is highly volatile, and in stark contrast with the desired stablecoin. What's more, NFTs can turn out to be worthless. These DLT applications must be properly regulated so that their uptake does not harbor an unintended factual barrier to abundance. Against this backdrop, the Ethereum platform recently introduced a new approach to proof of stake that addresses the immense energy wastefulness of traditional, legacy cryptocurrency mining (Ethereum's Big Switch to Proof of Stake, Explained | MIT Technology Review, n.d.). This example shows that implementing technical measures sometimes works better than creating new regulations.

#### Virtual and augmented reality

1. Virtual Reality can be described as a computer simulation that can be sensed and experienced by humans. It is a computer-generated simulation of a human sensory-perceived environment, which is usually three-dimensional (3D), visual, auditory and tactile. The simulation can be attained with the help of VR glasses or bodysuits, which make VR domains audible, visible and tangible for people. One is taken into a completely new, immersive reality. We are seeing more and more successful implementations of Virtual Reality (VR), Augmented Reality (AR), together referred to as Extended Reality (XR).

The practical applications of extended reality are myriad. XR is being implemented in industries such as education, healthcare (MRI scanners), processing trauma, ambulatory care, marketing (virtual try before you buy) and hyper personalized advertising, defense, sports, transportation, retail, product development (testing prototypes and data presentation), manufacturing (Digital Twin Technology) and editing, telecommunications and, of course, entertainment (metaverse, holograms), are no longer science fiction, but science fact. Entertainment and leisure are a category that will be broader and broader during the transition to the Age of Abundance, and will involve the tourism sector, recreational activities and outings, holidays and travel, art and culture, music and video experience, cinema, film and documentaries. The open-source platform High Fidelity, and Facebook's Metaverse are examples of real-time social VR.

2. The sky is the limit when optimized AI game algorithms will tap into the processing power of neuromorphic chips, memrites, 3D integrated circuits, optical computing and nano-biological computing. Neuromorphic CPU architecture is already being traded on the market in the form of human brain-inspired processors such as IBM TrueNorth and Intel LOIHI. This makes XR an abundance enabling set of technologies.
3. Concerning risks and legal certainty, the following pertains: rules must be introduced for the metaverse, which provide clarity regarding legal personhood and agenthood of people and virtual entities participating in this virtual space. In addition, the legal status of virtual property should be defined both in terms and conditions and end-user license agreements (EULA), and in law, preferably harmonized worldwide. Inspiration and lessons learned could be drawn from managing virtual worlds such as Second Life. Further, ethical issues such as perverse data harvesting, depersonalization disorder and embodiment techniques caused by virtual reality should be proactively addressed by a combination of technological measures, ethical VR by design, self-regulatory soft law instruments such as best practices and codes of conduct, as well as hard law (Slater et al., 2020)

Concerning the benefits: future societies should be structured around welfare promoting insights that current societies achieved in areas such as physical and mental health, creativity, social interaction, ethical standards, environmentally friendly product development, safety, and justice. Virtual reality (VR) offers an exciting glimpse into this utopian realistic vision of the future.

#### Hyper-accurate positioning

1. Advanced position technologies such as the Chinese BeiDou BDS-3 global navigation system can provide significantly more accurate results than their US predecessor GPS, reaching millimeter level precision positioning (Hyper-Accurate Positioning is Rolling Out Worldwide | MIT Technology Review, n.d.).^31^
2. Hyper-accurate positioning technologies will progress autonomous driving, precision agriculture, geological hazard monitoring (Ren and Yang, 2021).^32^ With that, it is a relative abundance enabling technology.
3. Hyper-accurate positioning techniques raise concerns about privacy and dual use. These should be addressed by the law, building on existing GPS and geostationary orbit (GEO) satellites regulations, and experience gained with matters surrounding territoriality, forum shopping and anti-spy protection. Further, this technology presents environmental questions concerning space rubble, for which best practices, etiquette, and space debris regulations in the form of international treaties should urgently be brought into practice.

##### Technological synergies

1. Cognitive computing

An interesting application in which a number of the above technologies are working together, is cognitive, neuromorphic computing: an innovative form of chip architecture. Cognitive computing is brain inspired computing. In cognitive computing, the morphology of the human brain serves as a source of inspiration for processors that perform computer tasks at high speed. With this goal in mind, scientists created computer chips that consist of a conventional Von Neumann architecture (or Princeton architecture) part on the one hand, and a neuromorphic part on the other. Neuromorphic chip architecture resembles the functionality of human left and right brain hemispheres.

2. Neuromorphic Chips

The Von Neumann portion of neuromorphic chips is particularly suitable for tasks traditionally associated with our left hemisphere, such as logic, analytical thinking and language centers. Examples of implementations of neuromorphic computing are NeuroGrid from the Stanford University Brains in Silicon project, as well as the Blue Brain Project. Both projects use an interconnected, parallel supercomputing hardware architecture. Finally, hybrid computing combines serial bits, artificial neurons and qubits. New computing paradigms such as cognitive computing, analog computing, optical computing and biocomputing are important drivers/enablers of innovative AI and quantum-AI hybrid systems, and thus enablers of abundance.

##### Regarding privacy

The question arises whether the benefits of this technology outweigh the relativization, or sacrifice, of certain aspects of privacy. In a long term, big picture vision, the good sides of new technologies, such as AI, should not be disproportionally negated by overemphasizing the importance of fundamental rights or—for that matter—the precautionary principle. Privacy preserving techniques aside, it is important to take stock with short intervals, and continue to make a cost/benefit trade-off. This involves a delicate balancing act. Moreover, it is conceivable that generations of people growing up with an Internet of Things (IoT) data trail will increasingly rate privacy less highly, when compared to competing fundamental rights such as freedom, mental and physical autonomy, and equality. Opting out from privacy and data protection—including data altruism (actively donating data) —are becoming increasingly popular, as data driven technologies grow to be ever more omnipresent. How privacy is valued is dynamic, contextual and culturally sensitive, as is ethics. Moreover, technology and privacy can be regarded as 2 sides of the same coin [*(2)* Desai, 2015; Towards Common European Data Spaces - EU Digital Policy Interview // CSBXL20 – YouTube, n.d.].

##### Regulation

From a regulatory perspective, the following applies to all technologies: generally, legislators should introduce universal, horizontal rules, in combination with vertical, sector-specific regulations that fit into an existing QMS. I envision an agile, technology, industry and sector specific horizontal-vertical ELSPI framework, equipped with a modern layered enforcement mechanism, which can adapt dynamically to changing societal needs and technological breakthroughs. The pictured enforcement mechanism commonly consists of a mix of self-regulation, soft law instruments and compulsory law (Kop, 2021e).

For instance, for AI, a bipartisan US AI Bill could be imagined as follows: as a softer version of the EU paradigm that would work in the US, constructed around a product liability system based on the pyramid of criticality, together with ex ante FDA-like certification/market authorization for hi-risk systems, products and services in the form of a US Compliance marking comparable to the EU CE-Marking, together with life cycle auditing, impact assessments and legal sandboxes for SME's that remove barriers to innovate. With the goal of remaining competitive internally and globally by building in democratic values, which, perhaps counter-intuitively, turns out to be just a few percent more costly for companies, compared to skipping the values.

A USA Compliance marking for hi risk AI systems, products and services is something companies will understand and adopt. Once adopted, such a framework could be tailored to other technologies such as quantum. The US and the EU should set global standards and the rules of the road together.

Attributing legal designations to these overlapping technologies and synergies will be a challenge, e.g., in quantum-AI hybrids, or nano-scale classical computing below 10 nm, in which quantum mechanical effects such as tunneling and energy quantization become unavoidable (Kop, 2021f). In this light, lawmakers should not hesitate to experiment with demarcating legal fields such as the Law of Quantum, and legal definitions such as the material scope of a quantum patent, to prevent codified law from falling too far behind the daily practice of technological advancements, which are taking place at an exponential pace (Aboy et al., 2022).

##### Technology impact assessment

In parallel to these legislative efforts, our democratic and distributed justice principles and values should be baked into the design, architecture and architecture of our applied technology. Useful tools that can help achieve these goals and guide the process, are industry specific technology impact assessments, implemented by multidisciplinary teams. These audits can also assist in realizing legal; compliance and regulatory conformity of exponential technologies, and should be done and/or updated at regular intervals, e.g., on a yearly basis. The tools themselves should also be updated regularly, and mirror our abundance society values, which are dynamic, context specific and culturally dynamic. An important responsibility for both scientists and entrepreneurs lies in actually using these tools and creating support for it internally,

Crucially, technology impact assessment should be implemented during all stages of technological development, including not limited to invention, innovation, and diffusion.^33^ The innovation process itself can be conceptualized as having various phases as well, such as idea generation and discovery, conceptualization and prototyping, commercialization, implementation, and follow-on invention (Friesike et al., 2015).

In addition, government coordinated technology assessment should be pursued, in the sense of systematically examining the effects of technology on society that occurs when a technology is introduced, analyzing intended and unintended consequences (Coates, 1976, p. 372–383). To this end the US Office of Technology Assessment (OTA) should be restored.^34^ After being reinstated, the OTA could offer informed, non-partisan policy recommendations on topics such as retraining the workforce, improving ethics, maintaining safety, and reducing inequality.

## Property, ownership and IP in the age of abundance

*Don't be afraid; from now on you will fish for people*.

*Luke 5:1-11*

The Bible quote in the heading of this section refers to an abundance of fish.^35^ The abundance was so overflowing, it almost sank the whole operation! Jesus claims no de facto, economic or legal ownership of the fish, no IP on his ways, as they are equally and bountifully distributed by his disciples under the populus. The Bible doesn't mention who owns the fish, nor IP on Jesus methods, trade secrets and know how. Are the fish public goods? Yes, essentially, Jesus made rival goods non-rival, at least temporarily (Adams and McCormick, 1987). Which might have caused a tragedy of the commons, and its wasteful overuse (Hardin, 1968; Rose, 1998). If Jesus fishing techniques were patentable, either IP would have expired, or he would surely have waived or pledged them into the licensed or public domain. His methods are probably still a trade secret. With Hume and Rawls, we could conclude that the Bible's authors did not believe these rights to be opportune, necessary, practical or justifiable, at least not on earth. But in a sense, it links abundance to equality and to good morality of sharing. Similarly, the Bible encourages people to focus on post-materialist virtues too, and on the art of living.

According to Fenell, property arrangements can be defined as socially rooted institutions that systematically organize and structure the conditions of resource access and usage (Fennell, 2013). This chapter views historic, contemporary and future property paradigms as stages in growth of social responsibility (Cf. Marx, 1972; Waldron, 2020).

In the above we concluded that our relative scarcity-based institutions should transform into technology driven relative abundance-based institutions. This metamorphosis directly affects economic and philosophical institutional justifications, as well as the practical design of legal concepts such as property and intellectual property and competition law. In parallel, technological progress is often at odds with the law, in particular property law, antitrust law and IP (Kop, 2020a, p. 328).

Addressing these challenges, it would be useful to have a broadened range of viable ownership governance structures in our toolbox, in the form of new modalities of property. It's all about legal and economic modeling of the ownership spectrum in the post-scarcity economy and the abundance society. In addition, we should critically examine the scope of artificially constructed scarcity through IP, and steer toward enrichment of the public domain.

This is not a matter of all or nothing: it requires a differentiated, refined approach. For example, completely abandoning IP rights in an online and offline setting is not recommended, as in that case the distributive justice benefits the less privileged groups of our world would disappear (Hughes and Merges, 2017). With that, copyright might have an equalizing effect on poverty. As Hughes and Merges showed:

*Copyright is, and can be, an important tool to promote a just distribution of income and wealth in society* (Hughes and Merges, 2017).

In addition, patents are an important recourse allocation mechanism in the health sector, where expensive clinical trials necessary during the stages of drug R&D would simply not be viable without the prospect of a return on investment. In this case too, society seems better off with IP.

But in a relative abundance setting it is quite possible that the above cases simply no longer exist.

Lessig too offers us a differentiated approach to IP in the information society, and considers a hybrid system that contains spaces free of IP, prompted by the nature of the internet (Lessig, 2004). For him, as well as for Boyle and Jenkins, the main theme is the reinforcement of the public domain, and the prevention of expanding IP rights and overprotection, for instance *via* compulsory licensing schemes, creative commons and open source software (Lessig, 2004; Boyle and Jenkins, 2018).

In the following, we carefully suggest perspectives, mindsets and ways of thinking on the content and design of modern property arrangements, which should possess a number of properties, or characteristics, that fit the post-scarcity economy. Individual and group access, underuse and overuse are important here. This applies to all types of resources, as well as to the 10 exponential technologies (their input, output and the tech itself, in the form of systems, products and services), precisely because these technologies are a driving force behind the transition to the abundance society. It is important that everyone thinks along about the direction we need to go.

Scholars should consider both ancient and modern forms of common, collective and private property and propose a socially equitable bundle of property rights tailored to the age of abundance. An ownership arrangement that connects property to liberty (and reward), and that decouples it from status and respect.

These modalities, these institutions must then be properly managed in a well-equipped governance system, which preserves the good things of capitalism and the market and combines them with the strengths of collective management. More about this in Section Equal relative abundance.

Policy makers should not be afraid to experiment with, common-pool resources (hybrid public-private goods), regulatory sandboxes, and declaring/categorizing primary resources such as data, as merit goods. Data in particular should not be monopolized, but distributed equally, and we should think about it not in terms of de facto economic or legal ownership, but in terms of access, rights and freedom to use.^36^

Back to property. Let me continue with the philosophical justifications for property,

According to Hobbes, and contrary to Lockean natural rights theory, property must be understood as the creation of the sovereign state (Cf. Marx, 1972; Hobbes, 1983). The normative political philosophies of both Rousseau and Kant prescribe that property has to be based on consent in the form of an agreement, or an hypothetical social contract, of everyone who will be impacted by decisions regarding how to utilize and govern a particular set of resources (Rousseau, 1968; Kant, 1991). On the one hand, this makes property a matter of social concern (Waldron, 2020, p. 15). On the other, it means that our social and institutional arrangements can be legitimized by democracy (Peter, 2021, p. 280).

In addition to these justifications, it is important to maintain awareness of the societal impact that a system of property entails. Against this backdrop, Waldron asks the following questions:

“*What overall model of community is generated by a given system of property rights and by the way they circulate in society?? What kinds of inter-personal relations does a given system of property foster? What ethos of economic interaction does it give rise to: an obsession with efficiency, an ethic of competitiveness, or a shared concern for those who are less well-off?”*

During the transition to the relative abundance society, these questions are especially relevant. As society shapes its institutions, our institutions shape society.

Moreover, according to Mill and Hume, property laws have never yet fully obeyed the principles on which the justification of private property—be it a social contract, one's labor, human personality, natural freedom, social responsibility, scarcity itself, or practices necessary to safeguard peaceful and secure possession of goods—leans (Mill, 1976, p. 14, 15; Hume, 1978; Rawls, 1999). But still, the systems cannot exist without their underlying principles, as these operationalize our values.

In light of the obvious unequal distribution of property across members of our society, it is up to us to create better alternatives to the existing systems.

Let us continue with a short overview of the types of goods that live in the property spectrum.

Ownership is usually organized into 3 types of property: state, commons and private (Heller, 2013). In addition, can we distinguish different categories of goods, such as: public and private goods, tangible and intangible goods, quasi-public goods, merit goods, club goods, and common pool-resources. Establishing this taxonomy of goods generally involves de concepts of rivalry, excludability and scarcity. For instance, information is often non-rivalrous in access and consumption and non-excludable in access and consumption, but not always.^37^ The classes of goods can be placed in a continuum of overlapping properties, where their characteristics are a matter of degree (Adams and McCormick, 1987, p. 192). Building on the trilogy of ownership, Heller makes a classification of 5 types of property: Open Access, Group Access, Private Property, Group Exclusion en Full Exclusion. Together, the author contends, these types reveal the full spectrum of property (Heller, 2013, p. 18, 19).

According to Heller,

“*Private property can no longer be seen as the end point of ownership. Privatisation can go too far, to the point where it destroys rather than creates wealth.”*

In this property spectrum, Hardin's notion of the tragedy of the commons (wasteful overuse of a common resource), and Heller's tragedy of the anticommons (wasteful underuse of a scarce resource), play a foundational role (Hardin, 1968; Heller, 2013, p. 6).^38^ Ostrom's common-pool resources can end, or prevent a tragedy of the commons, provided that not everybody acts for themselves:

*A common-pool resource is a hybrid between a public and private good in that is shared (non-rivalrous) but also scarce, having a finite supply* [Common-Pool Resource Definition, n.d.; (1) Sustainable Earth: Nobel Laureate, Elinor Ostrom, on How Can We Manage Common-Pool Resources – YouTube, n.d.].

According to Demsetz, property's main benefit to society as an institution is that it prevents overuse in a commons. Thus, exclusive ownership rights can prevent a theoretical tragedy of the commons in certain groups of resources. These types of goods should be paid close attention to by our good governors. According to Heller, we tend overlook cooperative solutions to overuse dilemmas (Heller, 2013, p. 11).

The notion of the tragedy of the anticommons makes visible the dilemma of too fragmented ownership beyond private property (Heller, 2013, p. 17). Too many owners can block each other from making any, or efficient use of a certain resource or good.^39^ While managing natural resources, our aim should be to find the sweet spot for property rights, between commons and anticommons (Heller, 2013).^40^ Access and exclusion dilemma's should be addressed by the state, by introducing legally structured group property forms that fit into the desired post-scarcity economy ownership spectrum, and hybrid rights that regulate access but put controls in place, resembling fishing quota (Heller, 2013).

We should investigate the usefulness of creating hybrid property regimes that allow people to bundle their ownership, and that allow the state, in certain cases, to expropriate fragmented rights (~eminent domain) to encourage access and cooperation. And yet be mindful of the many social and economic benefits that, according to Rose, ownership multiplicity and group access can have, pertaining to certain tangible and intangible goods (Rose, 2013). Empirical research should be done to elucidate anticommons effects per type of resource, per industry, and its effects on innovation. Thinking in terms of access and exclusion, freedom and rights, underuse and overuse, can help clarifying the options.

Now let us have a look at traditional IP justifications.

In general, philosophical perspectives that can justify property rights, intellectual property protection and antitrust rules, besides distributive justice, are the natural rights perspective, the personhood perspective, and the utilitarian, economic incentive perspective, which includes ensuring integrity of the marketplace (Menell, 2020).

There is a caveat though, in that property justifications do not always work well for intellectual property. This is due to the differences pertaining to possession of tangible goods, and the unphysicality of information and intangible ideas. For example, physical objects are more excludable, perceptible and rivalrous than ideas and emotions. This makes that the conventional economic justification for tangible property does not correspond well with intellectual property. IP is not a simple variation on the classic theme of property (Gervais, 2005).^41^ This is not surprising, given the historic fact that intellectual property as an institution did not exist when the canonical thinkers developed their justificatory theories for property.^42^

How would the doctrines that can justify intellectual property rights work for IP protection of exponential technologies? William Fisher canvassed 4 normative sources of intellectual property, which can be used to justify granting copyright protection from an economic, cultural and philosophical perspective (Fisher, 2001; IPTheories.map, n.d.; *William Fisher, CopyrightX: Lecture 1.1, The Foundations of Copyright Law: Introduction – YouTube*, n.d.). These are Welfare (including ensuring integrity of the marketplace), Fairness, Culture and Social Planning Theory. These normative sources do not apply easily to 4IR output, with humans increasingly out of the loop in the various upstream and downstream stages of the creative and inventive process, such as in Machine Made Creations and Inventions. Neither as a rationale for protection for the benefit of the AI Machine itself, nor the benefit of the AI Machine's programmer or the AI Machine's owner (Hughes, 1988; Fisher, 2001; IPTheories.map, n.d.; *William Fisher, CopyrightX: Lecture 1.1, The Foundations of Copyright Law: Introduction – YouTube*, n.d.). The same applies to automated quantum/AI hybrids outputs, and to any technological synergy as discussed in par 6 above, for that matter. Moreover, IP protection of the inputs and the systems themselves each can be questioned, making each of the justifications mentioned here problematic.

Let us now briefly zoom in on the economic incentives. In the utilitarian/welfare perspective, the public good problem needs to be solved. In this view, a public good like information that is non-rivalrous and non-excludable, will face underproduction without the IP incentive-reward mechanism. Meanwhile, competition lowers prices toward its marginal cost of production. Thus, government intervention is required to ensure adequate production that benefits society (Hughes, 1988, p. 18, 19). Limiting diffusion of knowledge *via* temporal IP monopolies that hinder cumulative, follow-on innovation, comes at a social cost though, especially in case of first-generation innovation. Heller writes this about IP and monopoly profits by exploiting intangible property in the information economy:

*To balance the values of innovation, disclosure, and competition, the US Congress keeps shifting the bundle of rights that a patent confers* (Heller, 2013, p. 23).

Moreover, first mover advantages and contracts could provide a sufficient return on investment while preventing imitation, making IP rights obsolete. With the market serving as the main engine of growth (Heller, 2013, p. 24). In an exponential innovation scenario, these points plus anticommons concerns could be economic arguments to at least shorten IP protection durations for the technologies described above. Risk assessment strategies could result in keeping technological breakthrough completely out of the IP realm (Brongersma, 2021, p. 17). Optimal IP durations can be measured by applying the Nordhaus model (Nordhaus, 1969, p. 3–7). The market, as a decentralized engine of progress is not always the best institutional choice to address the public goods problem (Menell, 2020). Hemel and Ouellette found that alternative incentive-reward mechanisms for allocating resources to inventions such as funding, grants, competitions and taxes, are better able to align economic incentives with social benefits, notably in healthcare (Hemel and Ouellette, 2019; Unboxing the Innovation Policy Toolkit with Professor Lisa Ouellette – YouTube, n.d.). In other words, the market is not always superior to central government planning.

Now let us discuss the limits of these justifications under post-scarcity conditions.

Canonical thinkers such as Hume and Rawls have suggested that property relations only make sense under conditions of scarcity (Waldron, 2020, p. 1; Hume, 1978). For Rawls, scarcity serves as a justification for liberal institutions like property and the market (Xenos, 1987, p. 239). Absent scarcity these justifications disappear, are less easy to uphold. But that's not the whole story, as discussions on how a resource should be used could be held, whether that particular good is scarce or not (Waldron, 2020, p. 1).

In general, the arguments of contemporary commentators criticizing the need for IP, such as Boldrin and Levine (2008), are stronger in a post-scarcity environment, than in a traditional economy.

### Intellectual property

The more relative abundance is present in a certain sector, the less IP overprotection can be justified. In addition, there is no tragedy of the commons in IP, but there can be a tragedy of the anticommons (Burk and Lemley, 2005, p. 1676). In the Digital Age, it not difficult to theorize about how an integrated mixture of IP forms causes IP overlap, IP thickets and with that a state of overprotection. In the case of holistic IP portfolio's, it is particularly important to have an integrated strategic understanding of the various IP branches, plus their fair competition and cybersecurity dimensions, instead of simply considering IP rights along doctrinal lines (Menell, 2020, p. 30). Even if empirical research result in evidence based findings pertaining to patents providing just the right incentives, all things concerned, it could still be that patents rights interplay with trade secrets and trademarks swing the pendulum to a state of overprotection, for a certain type of technology (Kop et al., 2022, p. 15, 16). Empirical research methods should take consolidated IP portfolio strategies into account, and study layered, simultaneous approaches to IP protection alongside “per IP right approaches”, especially in the case of complex AI and quantum infused machines.

### Property

As pertains to IP, the more relative abundance is present in a society, the less absolute, exclusive property arrangements can be justified, or are considered necessary from a practical viewpoint. This means more public domain, more common, public goods, and less enclosed, privately owned property. To make such as metamorphosis—which may seem quite radical at the moment—possible and politically, legally and socially feasible, we can learn from the Roman property paradigm. This is more layered, and (in theory) offers more sophisticated, tailored, sui generis solutions to the challenges ahead of us, especially in terms of access, excludability, commons, and public domain. A promising, understandable model that democratizes standalone AI output by a straightforward government issued public domain stamp, is res publicae ex machina (public property from the machine) (Kop, 2020a, p. 326–328).^43^

From a socio-economic, cultural perspective, we need a multilayered ownership arrangement that is capable of connecting property to liberty (and reward), while decoupling it from status and respect.

### Antitrust

From a business perspective ERA touches upon antitrust laws and winner take all effects, and the Schumpeterian view that temporal monopolies are necessary to ensure optimum levels of innovation. When searching for an innovation optimum, equality of opportunity, and more people able to participate in the innovation process, will probably compensate for winner takes all restrictions. I think that the winners would be allowed to have quasi monopolies, but should be forced by ERA regulations and morally compelled on the basis of post-scarcity values to give back to society (we should measure the effects on innovation, if possible). Moreover, Schumpeterian views will probably not work in post-scarcity economics. An ERA society needs revised fair competition laws, as these are based on legacy capitalism, antiquated economics, and outdated socio-economic values. Keeping the good things that work well of course!

### Solutions

Various solutions to the identified challenges pertaining to outdated (justifications for) property and IP arrangements have been suggested in literature.^44^

According (Heller, 2013, p. 23) to Lukas Peter, a commons theory of property:

“*would enable us to develop an understanding of property rights that is not based on exclusion, dominion and scarcity, but rather on access, democratic guardianship and relative, convivial abundance.”* (Peter, 2021, p. 143)

*Building upon the work of Locke, Rawls and Ostrom, the author recommends shifting our focus from productive capital and self-ownership to democratic guardianship of material resources held in common, while increasing individual freedom* (Peter, 2021).

According to Julie Cohen, in an informational economy, many kinds of resources might be managed as commons, which could ultimately lead to commons evolving into a property institution (Cohen, 2020).

“*the evolution of informational capitalism calls forth new propertization strategies and channels those strategies in particular (often very different) ways.”* (Cohen, 2020, p. 16)

According to the author,

“*The study of information property… demands a hybrid methodological approach that includes institutionalist, materialist, sociological and political economic lenses.”* (Cohen, 2020)

In the words of Benkler and Nissenbaum,

“*socio-technical systems of commons-based peer production offer not only a remarkable medium of production for various kinds of information goods but serve as a context for positive character formation.”* (Benkler and Nissenbaum, 2006)

Von Hippel postulates a free innovation paradigm, in which innovation commons such as free data and information together with open-source software and hardware strategies spur social welfare gains for all (von Hippel, 2016, p. 1; Potts et al., 2021). Unlike producer innovation, free innovation (by users and consumers) does not require intellectual property rights to function. Innovation commons are open innovation architectures without the need for IP. Free innovation theory is not about money but about human flourishing (von Hippel, 2016, p. 1).

Other commentators explore ideas for an inclusive, democratized knowledge economy, focusing on:

“*Transforming and disaggregating property rights so that different stakeholders—private or public investors, workers, local governments, and local communities—can make partial claims on the same productive resources.”* (Unger et al., 2019, p. 4, 5)

In a similar progressive vein, in an attempt to address the overlapping economic, social, and ecological crises humanity faces, the Democracy Collaborative advocates principles of democratic public control and ownership over IP and R&D, including:

*Moving towards a public knowledge commons approach to IP rooted in the principles of public ownership and equitable access;* and

“*Challenging corporations and monopoly power by linking public ownership and control of IP and R&D with efforts to increase competition in various economic sectors and diversify the ownership structure of enterprises and services (including cooperatives, publicly owned enterprises, and sustainable local and regionally based companies)*” (Hanna et al., 2020).

In conclusion, societies institutions have to change from incremental and fine-tuned, to radical and abolished. Otherwise, they will lose both social function and public support. But before we can give precise content to that, we must revisit the foundational principles underlying our institutions, in which our liberal democratic values are embedded. Are these principles ageless and abundance-proof no matter the context, or should both our principles and values change or be clarified, adapting to the times? Can we find answers in updated principles of distributive justice that build upon Rawls's thinking? We address this exciting question in the next section.

## Distributive justice

Principles of distributive justice can offer moral guidance for the political and institutional processes and frameworks that influence the distribution of burdens, gains, responsibilities and risks across society. These normative principles are associated with good governance in the sense that they can provide governments with both philosophical and economic justifications, and practical socio-economic arguments for legal, institutional and policy reforms.

According to Lamont and Favor,

*The economic, political, and social frameworks that each society has—its laws, institutions, policies, etc.—result in different distributions of benefits and burdens across members of the society* (Lamont and Favor, 2017).

Likewise,

*Every society has a unique mutual relationship of sources of law, such as the constitution, general laws, treaties, case law, customary law and general principles of law. The hierarchy of legal norms, standards and their interpretation and enforcement determines whether a particular legal concept or rule of law leads to the desired outcome* (Kop, 2020b, p. 16).

Consequently, the same distributive justice standard could be qualified as equitable and fair in a specific institutional context, or social order, and less reasonable in another context (Pieters and Demarsin, 2019). In addition, the internal separation of legislative, executive, and judiciary powers as commanded by Montesquieu's *trias politica* (Montesquieu, 1748) results in equivalent distributive justice principles having a different impact in different countries (Kop, 2020b, p. 16). This means that distributive justice principles could lead to different outcomes Europe and the United States, as opposed to China or Africa.

### Scope and role of distributive justice principles

Distributive justice concerns the socially equitable allocation of public and private resources, with a focus on the outcome and consequences of that distribution. Distributive justice principles have many aspects, and vary along different dimensions. Equal, proportional and fair distribution with regard to primary and secondary resources and necessities of life to members of society, such as income, tax, health, opportunities, and education, can be measured on a regional, national or global level, and across generations. As the distribution of these parameters is in constant flux, governments face continuous choices about how the distribution should be organized, which individuals or groups should be the recipients, and on what basis the distribution should be made, to ensure desirable and efficient outcomes (Lamont and Favor, 2017, p. 1).

Translating philosophical principles into concrete policy recommendations is an ongoing effort and involves complex methodological questions, including managing social expectations (Lamont and Favor, 2017). Technological breakthroughs, societal demand, gained insights or improved measurement techniques may give rise to updating and redefining the principles themselves, and reassessment of their practicality in their various dimensions.

### Families of distributive justice principles

Various types, or families of distributive justice principles have been developed over the past centuries. Often, these schools of thought run along ideological lines. Categories that may be relevant to the topic of this chapter, are, functional economic utility, or utilitarian/consequentialist-based Welfare principles,^45^ Egalitarian Principles, Libertarian principles, Feminist principles, Equality of Opportunity and Luck Principles, Desert-Based Principles, and the liberalism inspired Difference Principle.

We are not focusing on the Welfare Principle here, because -while the application of this principle may have led to progress in recent decades—it did not result in a balanced, equitable distribution of resources, and because utilitarianism fails to take seriously the unique characteristics of persons (Rawls, 1971).

In the words of Lamont and Favor,

*The challenge for contemporary utilitarians is to explain, given the massive informational requirements of utilitarianism and our apparent human inability to meet those requirements, how the population, and its experts, can plausibly arbitrate between conflicting policy and institutional recommendations coming from utilitarian theorists who share the same underlying normative principle* (Lamont and Favor, 2017).

### Rawlsian difference principle and desert-based principles

In this section we do concentrate on the Rawlsian Difference Principle, and Desert-Based Principles.

The Difference Principle as developed by Rawls aims to establish a lower limit in the quality of living conditions of all people (Rawls, 1993). Starting point is that everybody should have basic rights and liberties. Society's fundamental institutions should be arranged so that the distribution of primary goods, or basic needs, is to the maximal advantage of the average member of the least privileged social class (Menell, 2020, p. 13). Rawls' Difference Principle allows for deviations from strict equality as long as the inequalities in question result in the least advantaged in society being materially better off than they would be if absolute equality were maintained (Lamont and Favor, 2017, p. 1). This way, socio-economic inequalities should be addressed. The Difference Principle is bounded by a principle of equal opportunity (Lamont and Favor, 2017). As the difference principle benefits the poor, it should be applied to address the poverty paradox as described in paragraph 5 above.

Desert-Based Principles are formulated on John Locke's natural rights perspective that the labor of one's body and the works of one's hands gives a person ownership rights to these works. Put differently, people deserve to possess the products they make: the fruits of their labor become their exclusive property (Locke, 1988). Desert based principles advocate an initial fair distribution of resources, but tolerate inequalities of wealth resulting from the value of their productive contribution, effort in their work activity, sacrificing their time, risks taken through entrepreneurship and compensations of costs incurred during the appliance of their abilities, skills and talents (Lamont and Favor, 2017, p. 17). Desert-based Principles alone are not sufficient to ensure a socially equitable allocation of public and private resources, as people's productivity is influenced by many factors over which they have little control (Lamont and Favor, 2017, p. 18). They can however be used to articulate the Rawlsian Difference Principle.

### Normative parallels between exponential technologies and abundance

In paragraph 7 above, we determined that de normative sources of justification of IP rights are weak in the setting of AI generated creations and inventions (Fisher, 2001, p. 1–8). This logic can be applied to adjacent 4IR technologies as well, such as quantum computing. These normative sources belong to the same families of distributive justice principles. From a socio-economic, cultural and philosophical viewpoint, rationalizing, explaining and defending exclusionary rights (claims) on foundational 4IR technology, be it property or IP, is increasingly problematic, as abundance conditions increase. Therefore, also from the perspective of principles of distributive justice, we can see parallels between exponential technologies and abundance.

### Economic theory and distributive justice

Economic theory and distributive justice are intertwined. The distribution of burdens and gains across the population has an obvious economic dimension. While both normative and positive economists usually look at utility as their fundamental moral concept, philosophers employ a broader range of moral notions (Lamont and Favor, 2017, p. 7). For example, Rawls works from within a model of economic scarcity and an authoritative basis for the allocation of primary goods, to ensure social order and advance a concept of justice (Xenos, 1987). He connects the concept of moderate scarcity to distributive justice. Traditional economic theory alone, should never be enough to direct governance choices. To address post-scarcity conditions, we have to take this approach a step further: In a relative abundance society, policy decisions should be informed by positive post-scarcity economics insights accompanied/enriched by post-scarcity distributive justice theories/arguments.

Insofar as the distributive justice principles already function under traditional, scarce economics conditions, they in any case do not work under post-scarcity conditions. Conceptually, this has to do with the outdated kind of economics these principles are entangled with, as well as the values and ideals underlying the principles, such as morality and virtue, which were different in the context of relative scarcity. And with the nature of the socio-economic problems that had to be solved through application of the principles, directly or indirectly. For instance, liberalism is based on scarcity, not on sufficient abundance. Moreover, whether they have been applied adequately or not, the principles have not led to the desired equitable distribution of primary and secondary resources, with the above list of the 10 poorest countries in the world as an illustrative poverty paradox example.

In paragraph 2, we concluded that: “*Our institutions were built on the basis of preindustrial scarcity economics, and must now evolve into institutions based on abundance economics. Whereas, economics should be redefined, so do our institutions.”*

The same reasoning applies to the distributive justice principles that underlie our institutions: they must be redefined or modernized.

## Equal relative abundance

*Logic clearly dictates that the needs of the many outweigh the needs of the few*.

*Spock, Star Trek, The Wrath of Khan (1982)*

Conceptually, the conclusion is clear: the distributed justice principles ought to be synchronized with the properties of the abundance society. The theories should be updated to provide useful answers within the context of our new reality. Even though the transition from scarcity to abundance usually occurs gradually and incrementally, conceptually the new context is often at odds with the old circumstances, or radically/diametrically opposed to it. We literally stand with one foot in scarcity (conceptually, institutionally, values, economics, certain industrial sectors) and the other in abundance (this project/book, 4IR), which demands for mixed strategies. This irrevocably entails that we must further develop and rework the principles, or design a completely new principle. Building on lessons learned from the application of the various principles and their normative justifications over the centuries, including the Rawls difference principle. The difference principle, with its paradigm of equal opportunity combined with a lower threshold of material and immaterial primary goods—as well as its criticism in the form of the desert principle—is a promising candidate because it is a good theoretical starting point to address the poverty trap. Put differently: the world needs a new principle of distributed justice pertaining to relative abundance, which can help us solving the poverty trap/paradox on a global level.

This leads to the introduction of a new principle that connects abundance to equality. I would like to name it the Equal Relative Abundance (ERA) Principle of Distributive Justice.

### An equal relative abundance principle of distributive justice

We could imagine ERA as follows:

First and foremost, ERA builds on the Rawlsian difference principle, uniting desert-based critique on that principle into a Post Rawlsian principle of distributive justice with built in distributed equity, which makes sense in a post-scarcity environment. Crucially, ERA integrates desert-based principles to the extent that some may deserve a higher level of material goods because of inequality in contributions, i.e., their hard work, productivity, talent, luck or entrepreneurial spirit, only to the extent that their unequal rewards do also function to improve the position of the least advantaged.

This means, for instance, that we would still have a property system in which those who shoulder the burdens of prudence and productivity can hope to be rewarded for their virtue that separates them, to a certain degree, from those who do not, but these rewards cannot be completely internalized.^46^ This solidarity is required in order to reach the point of basic needs/relative abundance for the less advantaged social class, addressing the poverty trap, which will benefit society at large. Redistributive taxation, such as high-income taxes could have the desired equalizing effect, bringing back balance without removing the economic incentives to perform and achieve. That way, income differences could have an equalizing effect (Cohen, 1992).^47^ We could discuss the allowed size of the inequalities, but what is crystal clear, is that these should be significantly smaller than nowadays.

Thus, implementation-wise I suggest a differentiated approach—with some exceptions that prove and confirm the main rule—given that ERA is sensitive to considerations pertaining to desert, entrepreneurial spirit and risk-taking, luck, responsibility, consequences, henceforth integrating the good parts of other distributive justice principles.^48^

### Regionally differentiated implementations

ERA will have to be implemented in a territorially differentiated manner too, during the transition to the abundance society. As lifting people from poverty in Europe is a different thing than achieving ERA in the US, applying equal relative abundance techniques in Asia and Africa each have their own specific challenges and dimensions. As we saw above in Section Distributive justice, the specific institutional context in either a trias politica (EU) or a system of checks and balances (US), the type of economic systems, the socio-political order, as well as the cultural norms and mores of a particular country or region affect the role that principles of distributive justice can *de facto* play, and influence (impact) the social outcomes that application of the principles will have in the short and longer term.

### ERA impact assessments

We should therefore start assessing the ramifications that ERA may have now. The goal should be to predict and anticipate its consequences as accurately as possible, partly from the perspective of proportionality and subsidiarity standards. ERA impact assessments and scenario roadmapping techniques can assist us with mapping out desired and undesired side-effects. These tools will allow us to make adjustments where necessary. In addition to an overarching vision, this requires customization, prototyping and experimentation.

### Evolved economics

Second, a new form of redefined, evolved economics that takes into account both relative scarcity and relative abundance conditions, should be incorporated in our novel distributive justice principle. As economic theory and distributive justice are interconnected, positive post-scarcity economics insights and arguments (intertwined with philosophical perspectives and justifications) should participate in ERA.

### Integrating post-materialist values

Third, we should coalesce contemporary and post-scarcity values and ideals into the ERA principle and discuss in a multidisciplinary setting how exactly ERA should be operationalized, in a relative abundance economic, social and political context, so that it becomes a suitable underlying (foundational, first) concept to govern society and our institutions. Think Star Trek's Prime Directive.

Relatedly, we should align forward-thinking abundance enabling property arrangements with our post-Rawlsian distributive justice theory. What's more, both tragedy of the commons risks and anticommons concerns should be analyzed and addressed, by applying industry specific ERA solutions.

### Operationalizing ERA

The key to operationalizing ERA lies in defining a lower limit, or threshold to relative abundance. This threshold will depend—especially at the beginning of the transition—on regional differences in adequate abundance which are directly linked to the technological development of that region. I am referring to defining a proper set of primary material and immaterial goods containing ingredients such as income, healthcare, education, life/work balance, opportunities, and self-expression. Perhaps prosperity, happiness or wellbeing covers both material and immaterial needs. In that sense, ERA also offers distributive equity and customization. In a later stage of the transition, this lower limit will become more and more equal on a global level, i.e., the same for all people on earth. Due to technological progress and interplanetary travel, this lower threshold will subsequently increase for all people. Possibly with differences in relative sustainable abundance not on a regional level, but on a planetary level, indicating there would more or less abundance on Earth than on Mars or compared to other Earth-like spheres in our Milky Way galaxy. This would give rise to a relative cosmic abundance principle of distributed justice. A principle that should be assessed in real time, on an interplanetary level.

Please note that, even when societies' post-scarcity institutions would be grounded in enlightened (upgraded), post-materialist values, it is important that our system does not entirely disconnect property from freedom and autonomy. Otherwise, we would have communism or some other form of authoritarianism, which this chapter does not aim to endorse.

### Reflective equilibrium

I recommend discussing together the content, scope, role, and formulation of the proposed ERA principle, in an interdisciplinary gathering of the minds, utilizing Rawls method of reflective equilibrium (Stanford Encyclopedia of Philosophy, n.d.). This method is a clear process for how to choose, evaluate and revise between the distributive justice principles.

Let us enrich these constructive moral intuitions with applied ethics and empirical measurements so that we can have meaningful data driven distributive justice discussions. This demands inter alia for hi-quality data on peoples believes of the function of ERA in governing society, plus data on the historic policy effects of applying the various distributive justice principles, plus data on abundance measurements as suggested in paragraph 5 above. That way we can determine if people are ready for it, aware of the consequences, and what's needed to make them more ready or willing to enable system change. The older people are, the less interested they may be in change (as their material needs have been largely fulfilled then). The young have the energy, the ambition, the drive, the incentives, but not the methodology, the worked-out plans nor the positions of power, as they are not at the wheel of society.

We want these foundational ERA discussions to be quantitative, datadriven in nature, mixed with theoretical, qualitative insights. We then have that data because targeted empirical research has been carried out and will be carried out. Ultimately, our theory should possess prescriptive, descriptive, and exploratory elements, grounded in well-established legal philosophical traditions, enriched by reproducible, real world empirical evidence. I image ERA to be a principle that can be measured, eventually in real time.

In general, it is important that systematic quantitative and quantitative research is carried out into the role and meaning of distributive justice principles in light of relative sustainable abundance. More specifically, multidisciplinary ERA group debates should inspire informed, evidence based post-scarcity policy and abundance governance strategies.

## A government system tuned for abundance

Well managed, sustainable relative abundance requires good governance. Good governance requires a government system tuned for abundance. When thinking about a government system tuned for abundance, we should reconcile social, economic and political theory.

What exactly is the function of the government?

The government's main purpose is to safeguard society from thievery, violence, and individual power excesses, as well as dishonesty and fraud in business and industry,—and to do so in a productive and cost-efficient manner—society desires government, law, and order. Government is required to resolve conflicting demands on natural resources, as well as to prevent pollution and environmental degradation.^49^

According to Gallarotti, ideologies and political markets impact macroeconomic outcomes and government spending (Gallarotti, 2000, p. 2). He illustrates how, in the twentieth century, market society and the night watchman state gave way to the prosperous society and the guardian state (Gallarotti, 2000).^50^

In the Western hemisphere, people tend to agree that democracy is the ideal political system (Rappeport, 2003, p. 36). Democracy in itself can legitimize government and its institutions. In a liberal democracy we are dealing with political ideologies, such as left, right, liberal, progressive, moderate, or conservative. These political ideologies, such as liberalism, are based on scarcity (Xenos, 1987, p. 225). In our current time, we continue to search for a liberal democracy that strives for a better world through positive sum games, with respect for human rights and fundamental freedoms (Rappeport, 2003, p. 36). The opposite of a totalitarian system such as an autocracy or technocracy (Kop, 2021b). During the Age of Abundance, democracy remains our leitmotif.

Questions we can ask ourselves against the background of the transition to relative abundance include: Will the post-scarcity economy call for a different democracy inspired political system? Yes, I think that the abundance society requires a consensus democracy with better distributed justice. Will the post-scarcity society necessitate a different economic system? Without a doubt, we require an economy that takes into account relative abundance. And should we strive for a different kind of resource management? Indeed, the world needs well managed, sustainable relative abundance, through ethical post-capitalism. Lastly, does tech driven partial abundance demand for a new social contract between citizens and the state? Certainly, current societal transformation requires a new tailor-made social contract based on technology driven Equal Relative Abundance (ERA).

### Capitalism as an economic ideology

Capitalism is not a type of society, such as a liberal democracy, but an economic ideology that sets rules for achieving growth and societal progress by accumulating capital. In a capitalist system, a key governance challenge is how to balance and mix free-market economics with collectivist government control. As a response to this challenge, various models of capitalism exist today: free market capitalism, state driven surveillance capitalism, rentier capitalism, post-capitalism, technoscientific capitalism (Birch, 2020).

In a capitalist system, the market is traditionally considered to be the most capable mechanism to allocate scarce resources efficiently.

In the words of Giddens,

“*Capitalism is a system of commodity production, centred upon the relation between private ownership of capital and propertyless wage labour, this relation forming the main axis of a class system. Capitalist enterprise depends upon production for competitive markets, prices being signals for investors, producers, and consumers alike.”* (Giddens, 1990)

The genesis of capitalism thus far took place in four stages: private, joint stock, casino, and whiz kid, each phase characterized by devoting less and less available capital carefully and conservatively to facilitating trade, and investment into profitable enterprises.^51^ From this it follows that there is something seriously (dangerously) wrong with contemporary capitalism.

Various forms of post-capitalism have been suggested, such as economic democracy, participatory economy, social knowledge economy, anarchism, socialism, the post work society and the post-scarcity economy. Many commentators see technology as the main driver of post-capitalism.

Giddens too wonders what lies beyond capitalism. According to Giddens, humanity should strive for a post-scarcity system, coordinated on a global level, taking us beyond the dilemma of free market vs. central control, and avoiding self-destruction either by technology or a major war (Giddens, 1990, p. 163).

According to Peter,

“*the concept of the commons can strengthen democratic practices and institutions by limiting or even overcoming the negative political, socio-economic and ecological effects of open and competitive markets.”* (Peter, 2021, p. 279)

Birch conceptualizes rentiership as a technoeconomic practice technoscientific capitalism:

*Rather than entrepreneurial strategies based on commodity production, technoscientific capitalism is increasingly underpinned by rentiership or the appropriation of value through ownership and control rights (e.g., intellectual property [IP]), monopoly conditions, and regulatory or market devices and practices (e.g., investment dispute courts, exclusivity agreements)* (Birch, 2020, p. 3).

According to Schumpeter, the dominance of capitalism will result in a type of corporatism and the promotion of anti-capitalist principles, particularly among intellectuals. In advanced capitalism, Schumpeter argues, the intellectual and social climate required for entrepreneurship to flourish will not exist; it will be superseded by socialism in some form (Schumpeter, 1950).

According to Lundvall and Johnson, the classic arguments to aspire socialism, are:

1. *Ending the exploitation of the working class;*
2. *Socializing the means of production;*
3. *Preventing economic crises and unemployment;*
4. *Planning for the future;*
5. *Building science-based societies* (Johnson and Lundvall, 2020, p. 2, 3).^52^

Many of these classic arguments remain valid during the post-scarcity economy.

### Democratic post-capitalism

What might a Government System Tuned for Abundance look like?

Without a central government body that equally distributes limited resources over its population, scarcity, or paucity, implies free market driven competition over limited resources, and potential conflict over who owns and exclusively controls what. While competition and property are commonly associated with freedom, autonomy, self-expression, creativity and innovation, rivalry over and ownership of limited resources can lead to unequitable outcomes such as winner-take-all effects and income inequality.

Proponents of the markets argue that in a complex society, there are a plethora of decisions to be made on how to allocate certain resources to specific production processes. In market economies, these decisions are made on a decentralized basis by individuals and firms, and although not perfect, such a system often works more efficient than any alternative. Yet, history has taught us that a completely privatized economy always results in groups that are left out, who are worse off in a privatized economy than in a socialist alternative (Waldron, 2020, p. 18). The fully centralized collective government management of resources is many times less efficient than market solutions. It has proven impossible for central agencies operating in the name of the community and charged with overseeing the economy as a whole to make optimal decisions concerning their distribution (Waldron, 2020, p. 18). Central planning has often resulted in economic stagnation. However, sub-optimal outcomes do not imply that the ideals underlying socialism are not valuable and worthy of striving for.

I feel that we should move away from laissez faire style capitalism toward a mix of the best of both worlds, call it a relative sustainable abundance system, that somehow transcends the free market-central planning dilemma per industry, per region and eventually worldwide, as long as it is democratic in its core. This includes developing new forms of regional, national and global governance, and, in the US, avant-garde state level initiatives.

We need to transplant the good parts of our contemporary dominant systems into a government system tuned for technology driven abundance. A system that is democratic at its core, as:

“*The race for AI dominance is a competition in values, as much as a competition in technology.”* (Kop, 2020b, p. 1)

Moreover,

“*Cyberbalkanization could result in two parallel worlds, each with distinct divisions regarding technology, trade and ideology. In practice, this implies two opposing ecosystems would exist, each using its own standards and architectures that are incompatible with one other.”* (Lemley, 2021; Kop, 2021b)^53^

In this light, we can—perhaps counter-intuitive—find inspiration from the good parts of the Chinese innovation system, which are compatible with our Western way of life including our participatory democracy, and combine these with the ancient institution of German regional development banking. Regional development banking is an important driver behind the *Wirtschaftswunder* and the continued strength of German Mittelstand industries:

“*the medium sized companies spread throughout the country providing high quality specialist products and services to customers throughout the world.”* (see text footnote 52)

Incorporating these approaches could result in:

“*a unique combination of central planning for the long term and decentralized experimentation in the short and medium term”* (Johnson and Lundvall, 2020, p. 17)^54^

These institutions will therefore be different from the institutions with which everyone has grown up, and thus are so familiar with. The reforms require flexibility and support.

What's more, we should learn from history and consider implementing measures inspired by the social New Deal programs of the 1930s that helped the United States recover from the Great Depression, such as the Works Progress Administration (WPA), enacted by President Franklin D. Roosevelt.

Another scenario is a global system in which planned economies with strong market features coexist with market economies that have substantial, social engineered (The Venus Project, n.d.) planning elements (Johnson and Lundvall, 2020, p. 20).^55^

While most commentators agree on the necessity of reforms, not everyone is equally optimistic about the feasibility of socio-political change. According to Peter,

“*democratic capitalism and its underlying state-market dichotomy is most likely quite incapable of institutionally adapting and solving the diverse social, economic and ecological problems that exist.”* (Peter, 2021, p. 281).

Yet it is urgent to start experimenting with prototypes of hybrid, eclectic systems that combine and integrate the best parts of acceptable, forward-thinking socialist (in the sense of strong social policies and managing certain resources centrally or collectively) and ethical post-capitalist paradigms, built on democratic politics. (We should learn from, and avoid neoliberal, Reaganist and Thatcherist policies that where important causes of the 3 main systemic problems). Such a system could be dubbed: “democratic post-capitalism.”

As society transcends to the Age of Abundance, capitalism needs to evolve as well.

### A social contract based on technology driven equal relative abundance

Lastly, does tech driven partial abundance demand for a new social contract between citizens and the state? Or between the young and the old, men and women, consumers and entrepreneurs?

I think yes. Our current hypothetical social contract is based on scarcity. Market relations, social institutions and associated patterns of social recognition cannot be justified absent scarcity (Xenos, 1987). To legitimize our new systems and to create support for them, consent and engagement are indispensable. Support and engagement strengthen the acceptance of the authority of the state over individuals and companies. This is particularly necessary, because instead of a retreating state characterized by a decrease in government involvement, we want an active state that supports good governance. An active state that can bring the task of realizing well-managed sustainable relative abundance and equality through responsible long-term planning to a successful conclusion. Such a government should have quality, productivity and service as its ideals, applied to its core functions pertaining to law, infrastructure and welfare.^56^ (A stronger state is also necessary to make it clear that countries, and not behemoth platforms, are making the world's rules of the road). All this can be achieved by entering into a New Social Contract Based on technology driven Equal Relative Abundance (ERA).

Contrastingly, in a post-scarcity economy where more than enough is produced to fulfill society's wants and needs, it is expected that the decentralized market decisions no longer have to be made in the same degree of complexity and quantities, which makes the case for socialism and a certain amount of centralized planning stronger. The latter with the aim of achieving more equality, or to achieve a stable equilibrium. Nonetheless, to realize widespread relative abundance conditions, the top dogs will have to be restrained through progressive antitrust rules. This along with more decentralized modes of production and innovation, as diagnosed by Benkler and von Hippel (Benkler and Nissenbaum, 2006; von Hippel, 2016). For by themselves they will not share their wealth and means of production with the least of our brothers and sisters.^57^

The transition (transformation) from scarcity to widespread relative abundance requires thorough revision of our principles of distributed justice, our institutions, and our government system, from a post-scarcity standpoint. In addition to a government system tuned for abundance, this passage necessitates modernization of the morals and standards underlying these principles, which should evolve into a post-materialist values-system.

## Post-scarcity values

Above we concluded that a mindset shift is required to tackle the 3 major system challenges that we as humanity face. These problems concern environmental, sociopolitical and economic macroscopic system trends. Second, we concluded that when our primary needs are fulfilled, there is more room for a shift toward post-materialistic values, and that this shift is also a characteristic of the transition toward a post-scarcity society. Third, because the transition to a relative abundance environment is driven by the 10 described exponential, 4IR technologies, it is important that we embed our values in the design, operation and infrastructure of these technologies. This is technically challenging, yet possible. But will we be able to agree on the content of the values, which must then be operationalized into concrete governance principles? After all, as society shapes technology, technology shapes society. Values, which are dynamic, contextual and culturally sensitive, should therefore be aligned as much as possible with society as we envision it. We will have to discuss that vision of the future, that horizon, in a multidisciplinary context, on a regional, national and global level. There is much work to be done in this area.

There is a danger in the ongoing datafication of humanity, and the associated utility thinking. While using technology such as social media, we internalize the technological values of efficiency and individualism within ourselves. The convenience of social media does not create solidarity, but undermines social cohesion, empathy and involvement. Moreover, social life is taking place *via* the Internet in a sterile space, and not in an analogous, physical space as it used to be. Technological progress does not equal societal progress. It leads to a pampered generation without perspectives, unaccustomed to discomfort and danger, with a strong sense of entitlement to material things, without perseverance, entrepreneurial spirit and survival instinct, with atrophied value registers, and ultimately to a loss of humanity. Within a few generations, techno moral change can lead to an irreversible process (Swierstra et al., 2009). Therefore, we have to go back to meaning and dignity, and create familiar, physical, touchable conditions. In parallel, it is essential to develop and actively pursue a catalog of techno-moral virtues. Such initiatives will benefit social cohesion, solidarity, altruism, welfare and wellbeing, as well as creativity and productivity.

The efficiency, convenience and market thinking in the platform service economy also undermines utilities, public space and infrastructure. People themselves have become the end product in today's technocracy.^58^ These Silicon Valley revenue models need to be overhauled, as we are clearly at a crossroads. Those with progressive worldviews will build alternatives and lead the way for others to follow, hoping it is not too late to turn the tide. System change requires a mind shift, a change of focus and perception. We should be able to freely move toward a state of mind that we want, in a society that we want. During relative abundance conditions, we must be able to choose from more than either state or market driven surveillance capitalism. In this context, ideological core values such as democracy, autonomy and freedom of action are of vital importance.

The tech has to be aligned with this set of values. With every development and diffusion of new technology, an impact assessment should be made of the consequences that its roll-out may have on society. That is more innovation-friendly than strict application of the precautionary principle. Even when dealing with unknown consequences and risks, such an approach is always better than letting things take their course.^59^

I now mention some interesting ideas from the literature that can fuel our discussions about the content, design and purpose of abundance enabling post-materialist values.

According to Keynes, we need to transcend the personal and societal values and preoccupations of capitalism, and focus on the art of living and on what it means to be human (Keynes, 1972; Chernomas, 1984). The shift to post-materialism requires a cluster of values that transcends materialism in the negative, perverse sense of the word.

In the words of Stillman,

*the emphasis the material and the economic represents a narrow view of humankind - its potentials and its culture*—*a narrow view that may presage continuing crises of individual psychic wholeness (or motivation) and institutional legitimacy* (Stillman, 1983, p. 309).

Thus, in addition to a mental shift as to a more spiritual and balanced set of lifestyles (Sadler, 2010, p. 234), adequate, sufficient relative abundance also requires a cultural shift, as the modern community's standard of adequacy is severely distorted by questions of status (Dugger and Peach, 2009, p. ix–x). The real challenge will be to decouple property, work and leisure from status and respect, as there will always be vanity, jealousy and envy. (This includes separating property completely from negative recognition, and disconnecting property to a certain extent from positive recognition and positive desert).

Here lies an important role for parents and education, as, according to Inglehart's socialization hypothesis, the youth is more susceptible and more willing to change (Inglehart, 1977, p. 8). After reaching adulthood, values, norms, values and principles are more or less fixed. Moreover, empirical research shows that the older one gets, the more materialistic one becomes. Therefore, system change will have to come from the younger generations. For example, we see that young people are much more concerned with solving the climate problem and feel much more responsible for the wellbeing of our planet than older generations. In the words of Sadler, powerful social movements should be set in motion, able to influence political decisions about the allocation of resources at all levels (Sadler, 2010). It is promising that, according to Inglehart's quantitative insights, behavior can change within a few decades. Behavioral change will be necessary, in an era of exponential innovation.

The Inglehart thesis links the shift to post-materialistic values in the post-scarcity society to Maslov's hierarchy of needs, or pyramid of motivation, where everyone strives for happiness and fulfillment (Hoffman, 1988). Self-actualization is at the top of the pyramid. Maslov also gives us a definition of the self-actualizing human: it is about realizing your full potential, as in the full development of one's abilities and appreciation for life. Maslow was essentially right in that there are universal human needs regardless of cultural differences.^60^

Hai-Jew also—in the context of post-materialism—speaks about self-actualization, and on how to self-transcend:

“*People have to necessarily be self-interested to some degree as a protection mechanism. Without that, they will be taken advantage of by those around them. And yet, absolute Darwinian selfishness without social cooperation also does not work. Huge socio-economic disparities can destabilize social systems, but very flat or non-hierarchical socio-economic systems seem to suppress individual creativity and innovations and entrepreneurial innovations, broadly speaking.”* (Hai-Jew, 2020)^61^

For Giddens, self-actuation in the relationship between the self and society, means finding the proper balance between opportunity and risk (Giddens, 1991).

With Maslow I find it important to focus on the positive, benevolent sides of people. But not everyone will devote their lives to self-fulfillment, charity, spirituality and the creative and useful arts, such as music, literature, painting, science and technology. Where people will spend their time on intrinsically motivated creation and production based on their passion. Because what are people going to do with all that new free time? There is a chance that the masses will get bored in a phase of abundance and turn against the government (Lemley, 2015b). That's why the Romans had *panem et circensis*. People with lots of free time may indulge in revolutionary or self-destructive behavior prompted by events such as the abolition of certain rights, such as property, or catalyzed by political ideologies and conspiracy theories. Under the influence of platform technology, and the desire of companies and their algorithms for unbridled growth, this has already happened recently in the United States. It's also possible that everyone spends most of their time in virtual reality, in the Metaverse. Or in the Matrix.^62^

Entrepreneurs therefore have a special responsibility: they must pursue an Apollonian attitude instead of the Dionysian, in which democracy and human rights are in the foreground, from the first line of code. Businesses and engineers should be responsible and held accountable for the technologies they develop [Nemitz and Pfeffer, 2020; *(3)* “*Prinzip, Mensch. Macht, Freiheit und Demokratie im Zeitalter der Künstlichen Intelligenz – YouTube, n.d*.]. Silicon Valley companies ought to adopt an Apollonian attitude in world view, corporate ideology, philosophy of life and art.^63^ (Kop, 2020a, p. 336).

“*With the apollonian, derived from the name of Apollo, the Greek god of the arts, one indicates everything that*—*compared to the dionysic in world view, doctrine and art—bears the characteristics of the static, balanced intellect and that which strives for size, order and harmony. It is an attitude on which reason, boundary and balance have their stamp.”* (dbnl, n.d.)^64^

Ethical values and normative preferences about how our society's institutions should be reimagined, and how societies should be governed, are dynamic, contextual and culturally sensitive, as our societies are constantly in transit (Kop, 2021f). At the moment, due to the diffusion of exponential technologies, the world is changing faster than ever before.

Take quantum technologies:

“*The resulting mathematical inequalities, mysteries and paradoxes, such as the uncertainty principle, quantum tunnelling, quantum teleportation, quantum randomness and indeterminacy, and the parallel universes/many worlds interpretation of quantum mechanics, are counterintuitive to the human experience. For future generations of people, quantum phenomena that seem implausible and contradict observed reality might become more well-known and familiar.”* (Kop, 2021f)

Concluding this section: the Equal Relative Abundance (ERA) principle of distributive justice shall carry within it evolved, altruistic post-materialist values. Values such as less status, less materialism, less consumerism, more meaning and solidarity, combined with a deep sense of responsibility for a better environment and caring for our planet earth. There remains room for high-achievers (desert-based principles), but the new values might instruct lowered threshold of levels of adequate abundance wealth (Rawls difference principle), after primary material and immaterial needs have been fulfilled. Even with higher levels of technological development.^65^ Partly for this reason, such a distributed justice principle should be better able to justify the then reformed 'abundance proof' IP and property arrangements—in line with our 4IR policy recommendations from paragraph 6—such as more public domain, more freedom, and more sophisticated modalities of property. At least in theory this should all fit together nicely and correspond with each other. Then we can start policy prototyping and implementing ERA inspired societal governance models.

## Enablers and barriers of abundance and equality

Advancing society to a state of widespread equal relative abundance requires systemic policy reforms that enable abundance and take away barriers. To this end, the chapter now lists 15 barriers and 15 enablers of abundance and equality, which should be read in conjunction one with the other.

Barriers to abundance and equality:

1. The poverty trap/paradox (Sadler, 2010, p. 141).
2. Climate change and pollution (Sadler, 2010, p. 149).
3. The political system and its institutions, including conservative thinking (Boulding et al., 1978, p. 13, 14; Chernomas, 1984, p. 1024).
4. Bad governance (Sadler, 2010; de Graaf and van Asperen, 2018).^66^
5. The social cost of inequality has led to a clear perception of social injustice, social exclusion, a decrease in productivity and health, an increase in violence, and the phenomenon that governance has become less focused on law-making and enforcing, and more occupied with income-redistribution, which is inefficient (http://www.theartofgoodgovernment.org/neconppp.html, 2022).
6. Zero sum games such as inequality, classism, nationalism, sexism, racism, and war (Dugger and Peach, 2009, p. xii).
7. Increases in wealth not spent on preserving the environment, bequest value and solving inequality, but on weapons systems (Sadler, 2010, p. 237).
8. Knowledge predation and other forms of Dionysian entrepreneurial behavior (Rikap and Lundvall, 2020, p. 1).
9. Unfair competition law including a lack of modern antitrust law enforcement mechanisms, and incumbents preventing progress and social reform.^67^
10. Artificial scarcity in the form of IP and monopolies.^68^
11. Anticommons risks pertaining to transformative technologies in the form of harmful resource underutilization resulting from fragmented ownership (exclusionary) rights such as patent thickets, (Burk and Lemley, 2005, p. 1676; Heller, 2013, p. 6; Aboy et al., 2022).^69^
12. Casino and whiz kid capitalism, characterized by rentiers and speculators blocking investments, (Cf. Marx, 1972; Chernomas, 1984, p. 1016; Birch, 2020, p. 3; http://www.theartofgoodgovernment.org/neconppp.html, 2022).
13. Negative externalities (Giddens, 1990).
14. Path dependency: the heavy hand of the past (Slijpen, 2017).^70^
15. Mother nature, human nature and our pre-abundance society values system (Keynes, 1963, p. 362; Chernomas, 1984, p. 1010).

Taking away the above-mentioned roadblocks will benefit the transition to a post-scarcity society.

Enablers of abundance and equality:

1. Solving inequality should have priority, by addressing the poverty trap (Sadler, 2010, p. 146),^71^ and redesigning society's institutions on the basis of the Equal Relative Abundance (ERA) principle of distributive justice.
2. Good governance carried out by a concerned, responsive government.
3. Translating the 4IR technology policy recommendations into concrete regulatory strategies.
4. Making properly designed technologies with post-scarcity values embedded into their architecture and infrastructure mandatory *via* standardization, certification, benchmarking and life cycle auditing.
5. Business collaboration (Sadler, 2010, p. 221), responsible entrepreneurship combined with an Apollonian entrepreneurial spirit as opposed to a Dionysian world view.
6. Universal employment, including increasing women's economic contribution by encouraging their participation in the labor market (Dugger and Peach, 2009). Universal employment is required to reach sufficient levels of relative abundance, while reducing the cost of lost opportunity.^72^
7. Employing German Regional Development Banking for Jobs and Productivity (see text footnote 55).^73^
8. Redistributive policies such as Universal Basic Income, taking from the 1% and sharing their wealth with the many (Brooks and Harter, 2021; STANFORD Magazine, n.d.).
9. Democratically structured common property arrangements that include rights to democratically regulate them, making possible the sustainable management of common property resources, and enabling people to develop and enforce rules and regulations against free riding and unlimited appropriation (Peter, 2021, p. 283).
10. Managing capitalist economies in such a manner that they preserve the welfare of workers (Gallarotti, 2000, p. 40).
11. Re-distribute gains in productivity to workers on the basis of ERA, instead of attributing profits to the happy few running the corporations, and the rentiers (http://www.theartofgoodgovernment.org/neconppp.html, 2022).
12. Strengthening elements of socialism in most societies, e.g., restricting and or restraining antiquated capitalist ownership of capital and natural resources, in tandem with radically changing current forms of outmoded political governance where nation states compete in attracting private capital and in protecting knowledge through enclosure (Johnson and Lundvall, 2020, p. 22).
13. Even though China is a systemic rival of the US and their ideology is incompatible with democracy, we must still be open to learn from Chinese poverty reduction by creating a knowledge economy, developing green, decarbonizing technologies, long-term planning in combination with decentralized experimentation, and more efficient, productive state control. Counter-intuitively, Western societies should not be afraid to transplant the acceptable, well-functioning parts from the Chinese approach that are compatible with the human rights and freedoms we cherish, into their own democratic, post-scarcity systems (Johnson and Lundvall, 2020). What's more, we should learn from history and consider implementing measures inspired by the social New Deal programs of the 1930s that helped the United States recover from the Great Depression, such as the Works Progress Administration (WPA), enacted by President Franklin D. Roosevelt (Thoughtco, n.d.).
14. Encouraging sustainable innovation plus accompanying IP models in developing countries that include waiving and pledging of IPRs on the basis of TRIPS flexibilities (Suthersanen, 2006).
15. Solving zero and negative sum games and pursuing positive sum games (Rappeport, 2003, p. 43).

Introducing the enabling policies mentioned above will propel the transformation to the relative abundance society.

The prospects of a system change to adapt to post-scarcity conditions do not always look good from a socially critical lens. This is caused, among other things, by a fear of change, vested financial interests, ideological differences, a lack of international cooperation, and human nature (Sadler, 2010, p. 231–237). Nonetheless, this book project's insights have value. In the words of Giddens,

“*all discussions which propose such possible futures, including this one, can by their very nature make some impact.”* (Giddens, 1990)

## Conclusion

The central thread through this chapter is the role of technology as an engine of change. Naturally, technology is not the primal cause for all our difficulties, nor is technology our only salvation. Technology evangelists should spread the word about the advent of an age of widespread relative abundance, and encourage people to think through its consequent macroscopic system challenges in inclusive, multidisciplinary settings. Let's change this world together!

This chapter views relative scarcity and relative abundance as temporal socio-economic categories at two opposite sides of a continuum. The chapter unifies good governance with equality and abundance, by introducing a post-Rawlsian Equal Relative Abundance (ERA) principle of distributive justice. Crucially, ERA integrates desert-based principles to the degree that some may deserve a higher level of material goods because of inequality in contributions, i.e., their hard work, talent, luck or entrepreneurial spirit, only to the extent that their unequal rewards do also function to improve the position of the least advantaged. A society governed by the ERA principle with built in distributed equity, should in theory be able to solve the poverty trap on a global level. It concludes that the strategic reforms necessary to balance the socio-economic effects of 4IR technology now, fit the trend of a shift from scarcity to well-managed relative sustainable abundance for all.

Principles should govern our actions. This chapter views historic, contemporary and future property paradigms as stages in growth of social responsibility. Society requires property arrangements that do not exacerbate the inequalities, but rather mitigate them, in line with ERA. We should actively embed our norms, standards, principles and context-specific values both in the design and infrastructure of our technology, and in our socio-economic, political and legal institutions. Although philosophers like Mill and economists like Demsetz have said that the practicalities of our institutions such as ownership and IP are not always in line with their underlying moral and philosophical justifications, plus the institutions will never be perfect in their consequences, I believe that the principles—which must be based on our agreed upon, evolved post-scarcity values—should form the starting point of our search for the best system.

Much work must be done in this area. We have to discuss the interpretation and scope of our operationalized principles and their foundational values—which are culturally sensitive—in inclusive, interdisciplinary groups, using qualitative and quantitative scientific methods. The outcomes and insights gained from datadriven, multimethod research should then inform concrete policy actions on a regional, national and global level. This is a dynamic, continuous effort that requires our combined thinking power, open mindedness and flexibility, in a solution-oriented spirit of cooperation.

An Age of Abundance requires a government system tuned for abundance. When thinking about such a system, we need to bring together social, economic and political theory, in light of the function and purpose of the state. The chapter posits that it is urgent to start experimenting with prototypes of systems that mix the best parts of acceptable, forward thinking socialist and ethical post-capitalist paradigms, built on participatory democracy. When searching for a post-scarcity synthesis of progressive, liberal democracy inspired capitalism and socialism that combines the best of both worlds, an important question remains who should (co)control vital resources and the means of production.

Perhaps counter-intuitively, this chapter advises to draw inspiration from the acceptable parts of the Chinese innovation system,—such as long-term planning in combination with decentralized experimentation—provided these elements correspond with our Western way of life (freedoms) and our participatory democracy. In addition, the chapter recommends implementing the ancient institution of German regional development banking, avoiding the limitations of traditional banking and ensuring quality, productivity, stability and economic growth. What's more, we should learn from history and consider implementing measures inspired by the social New Deal programs of the 1930s that helped the United States recover from the Great Depression, such as the Works Progress Administration (WPA), enacted by President Franklin D. Roosevelt.

Societal change starts with a purposeful vision: an ideal or a goal toward which one aspires. Without a clear vision driven by its underlying ideals, there cannot be a defined path toward meaningful destination.

## Data availability statement

The original contributions presented in the study are included in the article/supplementary material, further inquiries can be directed to the corresponding author.

## Author contributions

The author confirms being the sole contributor of this work and has approved it for publication.

## Conflict of interest

Author MK was employed by AIRecht. The handling editor ML declared a shared affiliation with the author at the time of review.

## Publisher's note

All claims expressed in this article are solely those of the authors and do not necessarily represent those of their affiliated organizations, or those of the publisher, the editors and the reviewers. Any product that may be evaluated in this article, or claim that may be made by its manufacturer, is not guaranteed or endorsed by the publisher.

## Footnotes

^1^From a socio-cultural perspective the Age of Abundance comes after the period known as Modernity.

^2^In this regard, the Kardashev scale creates a taxonomy that determines the level of technological advancement of a civilization or the basis of the amount of energy it is able to use and control, on a cosmic scale. A Type I civilization can be outlined as one that is able to harness all the energy that *reaches* its home planet from its parent star, which involves extensive application of fusion power, antimatter and renewable energy. Type II would be a civilization capable of utilizing the energy *radiated* by its own star, i.e., the Sun. Type III is characterized as a civilization that possesses energy at the scale of its own galaxy, in Earth's case the Milky Way. At present, humanity has not yet achieved Type I civilization status. See (*Kardashev Scale – Wikipedia*, n.d.).

^3^See also (Korotayev, 2006).

^4^The authors conclude that good governance necessitates good governors guided by benevolence.

^5^The 4IR refers to a new technological age in human history and comes after the Third Industrial Revolution, or Digital Revolution, which started in the 1970ties.

^6^Antipode is a concept from geography.

^7^A resource's natural limit is independent of demand. In a world that obeys our current laws of physics, time is infinitely scarce (Scarcity Definition, n.d.).

^8^According to these authors, the wants-needs distinction is a central element of the definitions of absolute and relative scarcity.

^9^This impulse seems strongly embedded in our DNA, especially in those working at Wall Street.

^10^Such as living habits and standards, norms and values. In the present context, these *mores* cannot be viewed separately from the degree of technological advancement and sophistication of a society.

^11^According to the authors, adequacy levels depend upon the knowledge context in which people operate.

^12^There are also thinkers who reject the pursuit of economic growth as an end in itself.

^13^In response to materialism, the author advocates a more spiritual or at least more balanced set of lifestyles.

^14^Research should be both theoretical and empirical.

^15^Veblen coined the term conspicuous consumption, referring to a public display of economic power and status goods.

^16^Keynes differentiates between needs of the first class and needs of the second class, the latter being ‘'those which satisfy the desire for superiority, may indeed be insatiable” (Keynes, 1963).

^17^Please note that the Earth is not the center of the universe.

^18^In the US, the Founding Fathers developed a system of checks and balances to *balance* legislative, judiciary and executive powers, with each institution both controlling and cooperating with the other two powers, instead of completely *separating* them into independently operating pillars as proposed by Montesquieu. Both ideas aim to guard individuals from a concentration of state power (Smismans, 2002, p. 94).

^19^With China being the exception to the rule, although there are big differences between living standards in rural areas and the city.

^20^*See also*: Rafikov and Akhmetova (2019) writing about “the paradox of scarcity in the age of abundance” and Sadler, *supra* note 2, at 52. According to Sadler (2010), resolving the poverty trap must lie at the heart of a successful transition to a post-scarcity global economy. The transition itself poses many challenges, the main one being scarcity replicating itself while people's ability to pay for things decreases, in parallel with cost-reducing innovation. A solution might be to tax capital more than labor.

^21^Another noteworthy method to characterize and manage technology emergence is the concept of technological speciation, based on semantic patent analysis (Moehrle and Caferoglu, 2019).

^22^According to the authors, a multimethod strategy will be required to bring the problem of scarcity and abundance under intellectual and policy control (Boulding et al., 1978, p. 8, 14).

^23^*To humans forec*asting exponential growth is counter-intuitive, as we are tuned for Darwinian paced, linear trends, *see*, Mark Michaelis, [Exponential Technologies], Preparing for the Exponential Technology Revolution (Microsoft Docs, n.d.).

^24^Another promising method to ensure responsible AI is symbolic reasoning (The New Version of GPT-3 is Much Better Behaved (and Should be Less Toxic) | MIT Technology Review, n.d.).

^25^Although this is ground-breaking science, self-replication clusters of cybernetic cells could benefit from some ethical considerations in combination with innovation controls using regulation and risk management tools (Kriegman et al., 2021).

^26^Furth reading on Star Trek and the Abundance Society (The New York Times, n.d.).

^27^An opinion of law or necessity is the conviction, or judicial perspective that an action was carried out because it was a legal obligation, a judicial tradition, or a customary right.

^28^For a discussion of anticommons concerns relating to quantum computers, see (Kop et al., 2022).

^29^According to the authors, “the structure of the biotechnology industry seems likely to run high anticommons risks.”

^30^Proposing a copyright infringement exception for 3D printing.

^31^The Chinese BeiDou and Russian Glonass are currently being integrated to form an alternative for the US GPS.

^32^In addition to meter-level standard positioning services, BDS-3 features precise point positioning (PPP) and real-time kinematic (RTK) positioning technology.

^33^For further reading on the nature of technological development (Schumpeter, 2006).

^34^*See for an overview of its history* (Sachs, 2022; Brookings, n.d.).

^35^This recite has three meanings. It refers to one of the first known environments of temporal, regional relative abundance in human history. Second, it symbolizes the beginning of a new era. Third, people should not be afraid of change.

^36^Privacy and data protection aspects of data being an exception to this rule.

^37^See for a critical discussion of the idea that information is a public good since it is non-rival and non-excludable as a justification for IP as an institution (Ouellette, 2015).

^38^For a critique of the notion of the tragedy of the commons (Rose, 1998; Ostrom, 2015).

^39^Music IP clearances are a good example of wasteful underuse caused by fragmented exclusionary rights, with myriad copyrights and related rights holders blocking each other from efficient use of a particular composition or recording. A similar tragedy of the anticommons exists in machine learning training datasets with multiple owners potentially blocking data sharing.

^40^*Cf*. (Parisi et al., 2005) diagnosing a misalignment of private and social incentives in common resource scenarios, and endeavoring a dual property system that regenerates the natural conformity between use and exclusion rights, while taking into account externalities.

^41^For further reading on the relation between IP and trade (The Political Economy of Intellectual Property Law, 2004).

^42^See also (Lemley, 2015a) *Cf*. (Hughes and Merges, 2017, p. 6).

^43^*See* par 6.I above under Artificial Intelligence. Creative Commons CC0 form would have the same effect for copyright.

^44^We leave libertarian critique behind in this place.

^45^Jeremy Bentham is considered to be the father of Utilitarianism (Bentham, 1948).

^46^Cf. (Munzer, 1990).

^47^In a sense, this is the opposite of trickle-down economics.

^48^For views that can be used to articulate the ERA principle, *see*, effort, sacrifice and desert (Hettinger, 1989, p. 37–42); risk, effort, skill, luck (Wilson, 1993, p. 73–76); reward according to contribution (Miller, 1989).

^49^http://www.theartofgoodgovernment.org/index.html (2022).

^50^At the moment however, parts of the free world experience a democratic recession. AI might be one of its causes.

^51^http://www.theartofgoodgovernment.org/neconcap.html (2022).

^52^Other arguments are *Gender equality, anti-imperialism, giving workers access to the fruits of culture (literature, art, theater, music) and generally improved living conditions* (Johnson and Lundvall, 2020, p. 5).

^53^Discussing the idea of the Splinternet.

^54^http://www.theartofgoodgovernment.org/Xecon1devbank.html (2022).

^55^Compare to: (Lemley, 2021).

^56^http://www.theartofgoodgovernment.org/costeff.html (2022).

^57^This calls for an Apollonian entrepreneurial attitude.

^58^Banning profiling cookies can contribute to this goal.

^59^For further reading about policy dilemma's as regards the societal impact of emerging technology (Genus and Stirling, 2018); For further reading on technology impact assessment (Sachs, 2022, p. 48–51).

^60^*Cf*. (Sadler, 2010, p. 30). Sadler is less optimistic as regards the feasibility of an actual change towards postmaterialistic values and lifestyles: “*The engines that drive economic growth – new scientific knowledge, technological change, human ingenuity and the desire for improvement in living standards are too powerful.”* (Sadler, 2010, p. 234).

^61^Post-materialist self-actualizing is not to be confused with extreme ascetism.

^62^Are we living in a simulation?

^63^Terms, introduced by Nietzsche (1844–1900) in his Die Geburt der Tragödie aus dem Geiste der Musik (Nietzsche, 1872), and inspired by the philosophy of A. Schopenhauer (1788–1860).

^64^With regard to the arts, the term “apollonian” refers to light and comprehensibility, reason, symmetry, beauty and healing. According to Nietzsche, neither the apollonian nor the dyonisian ever prevails, due to each containing the other in an eternal balance (The Birth of Tragedy – Wikipedia, n.d.).

^65^As explained earlier, higher levels of technological development demand higher levels of adequate abundance. In other words, lower levels of technological development require lower levels of relative abundance, i.e. that abundance is sufficient sooner under these less technologically advanced conditions.

^66^Six indicators of bad governance used by the World Bank are: (1) voice and accountability, (2) political stability, (3) government effectiveness, (4) regulatory quality, (5) rule of law, (6) control of corruption (The World Banks, n.d.).

^67^Commentators showed us that both IP laws and antitrust laws prevent the transition to a post scarcity economy and with that the advent of the abundance society, (Burk and Lemley, 2005, p. 1676; Desai and Magliocca, 2014, p. 1698; Mehra, 2016, p. 6, 39; Kop, 2020a, p. 336; Aboy et al., 2022).

^68^The scope of copyright, patents, trade secrets, trade name and trade dress rights should be limited and balanced with fair competition. For instance, by raising the creativity bar and expanding exceptions and limitations (Suthersanen, 2004).

^69^Anticommons problems in new technologies such as quantum, biotech and mobile phones should be avoided.

^70^Path dependence is the process by which past events or choices affect the course of later developments, especially because certain options become difficult or precluded. History plays a prominent role in path-dependent processes. After all, where we go is highly dependent on where we come from. In public policy, present policy choices are constrained by institutional pathways that emanate from choices made in the past. Path-dependent processes pose important barriers to the pursuit of widespread relative abundance.

^71^For causes and cures of extreme poverty.

^72^Please note that more empirical research on the socio-economic effects of universal employment in conjunction with worldwide universal basic income on abundance is required.

^73^Implementing the ancient institution of German regional development banking avoids the limitations of traditional banking and ensures quality, productivity, stability and economic growth.
